# Anthrax Toxin Receptor 2 Functions in ECM Homeostasis of the Murine Reproductive Tract and Promotes MMP Activity

**DOI:** 10.1371/journal.pone.0034862

**Published:** 2012-04-17

**Authors:** Claire V. Reeves, Xing Wang, Pelisa C. Charles-Horvath, Joy Y. Vink, Valeriya Y. Borisenko, John A. T. Young, Jan K. Kitajewski

**Affiliations:** 1 Department of Obstetrics and Gynecology, Columbia University Medical Center, New York, New York, United States of America; 2 Department of Pharmacology, Columbia University Medical Center, New York, New York, United States of America; 3 Nomis Center for Immunobiology and Microbial Pathogenesis, The Salk Institute for Biological Studies, La Jolla, California, United States of America; 4 Department of Pathology, Columbia University Medical Center, New York, New York, United States of America; 5 Herbert Irving Comprehensive Cancer Center, Columbia University Medical Center, New York, New York, United States of America; Ecole Polytechnique Federale de Lausanne, Switzerland

## Abstract

Anthrax Toxin Receptor proteins function as receptors for anthrax toxin, however physiological activity remains unclear. To evaluate the biological role of Antxr2, we generated *Antxr2*−/− mice. *Antxr2*−/− mice were viable, however Antxr2 is required for parturition in young females and for preserving fertility in older female mice. Histological analysis of the uterus and cervix revealed aberrant deposition of extracellular matrix proteins such as type I collagen, type VI collagen and fibronectin. A marked disruption of both the circular and longitudinal myometrial cell layers was evident in *Antxr2*−/− mice. These changes progressed as the mice aged, resulting in a thickened, collagen dense, acellular stroma and the disappearance of normal uterine architecture. To investigate the molecular mechanism underlying the uterine fibrosis we performed immunoblotting for MMP2 using uterine lysates and zymography using conditioned medium from *Antxr2−/−* mouse embryonic fibroblasts and found reduced levels of activated MMP2 in both. This prompted us to investigate MT1-MMP status, as MMP2 processing is regulated by MT1-MMP. We found MT1-MMP activity, as measured by MMP2 processing and activation, was enhanced by expression of either ANTXR1 or ANTXR2. We identified an ANTXR2/MT1-MMP complex and demonstrated that MT1-MMP activity is dependent on ANTXR2 expression levels in cells. Thus, we have discovered that ANTXR1 and ANTXR2 function as positive regulators of MT1-MMP activity.

## Introduction

The Anthrax Toxin Receptor (ANTXR) proteins, ANTXR1 and ANTXR2, are cellular receptors that contain a von Willebrand factor type A (vWF) domain, a transmembrane domain and a cytosolic tail with putative signaling motifs. vWF domains are known to facilitate protein-protein interactions when found on extracellular matrix (ECM) constituents or cell adhesion proteins like ∝-integrin subunits [1] and constitute ligand binding sites on ANTXRs [2]. Both ANTXR1 and ANTXR2 have been demonstrated to interact with ECM proteins *in vitro* [3], [4], [5]. The proteins also bind anthrax toxin, however, the *ANTXR* genes were originally identified based on expression in endothelium suggesting a physiological role in angiogenesis. Consequently, many groups have explored ANTXR function in endothelial cells. We demonstrated that ANTXR2 is required for angiogenic processes such as endothelial proliferation and capillary-like network formation *in vitro* [6]. Similarly, ANTXR1 has been demonstrated to be important for endothelial cell migration and network formation [4], [7]. Despite these studies, the physiological function of the ANTXR proteins remains to be fully elucidated.

A role has been proposed for *ANTXR2* in ECM homeostasis [5], [8] based on ANTXR2 protein structure and ECM binding capability. *Antxr1*−/− mice exhibit defects in ECM deposition in organs such as the ovaries, uterus, skin, teeth and skull [9]. Furthermore, a rare human disease is caused by mutations in the *ANTXR2* gene. Systemic Hyalinosis is an autosomal recessive disease that encompasses two syndromes, infantile systemic hyalinosis (ISH) and juvenile hyaline fibromatosis (JHF) [8], [10], [11]. ISH and JHF are characterized by gingival hypertrophy, progressive joint contractures, osteolysis, osteoporosis, recurrent subcutaneous fibromas, and hyaline depositions which are thought to form as a result of abnormal collagen and glycosaminoglycan accumulation [12].

To investigate the physiological role of Antxr2, we disrupted the gene and discovered that *Antxr2* is not essential for normal development, but is required for murine parturition in young pregnant mice and for preserving fertility in aged female mice. Histological analysis of the uterus and cervix revealed aberrant deposition of ECM proteins causing severe disorganization of the cellular composition of these tissues. We investigated the molecular mechanism behind these defects and discovered that ANTXR2 is a positive regulator of MT1-MMP activity, a key protein that activates MMP2 and functions in ECM turnover.

## Results

### *Antxr2*−/− Mice Exhibit a Failure in Parturition

**Figure 1:**
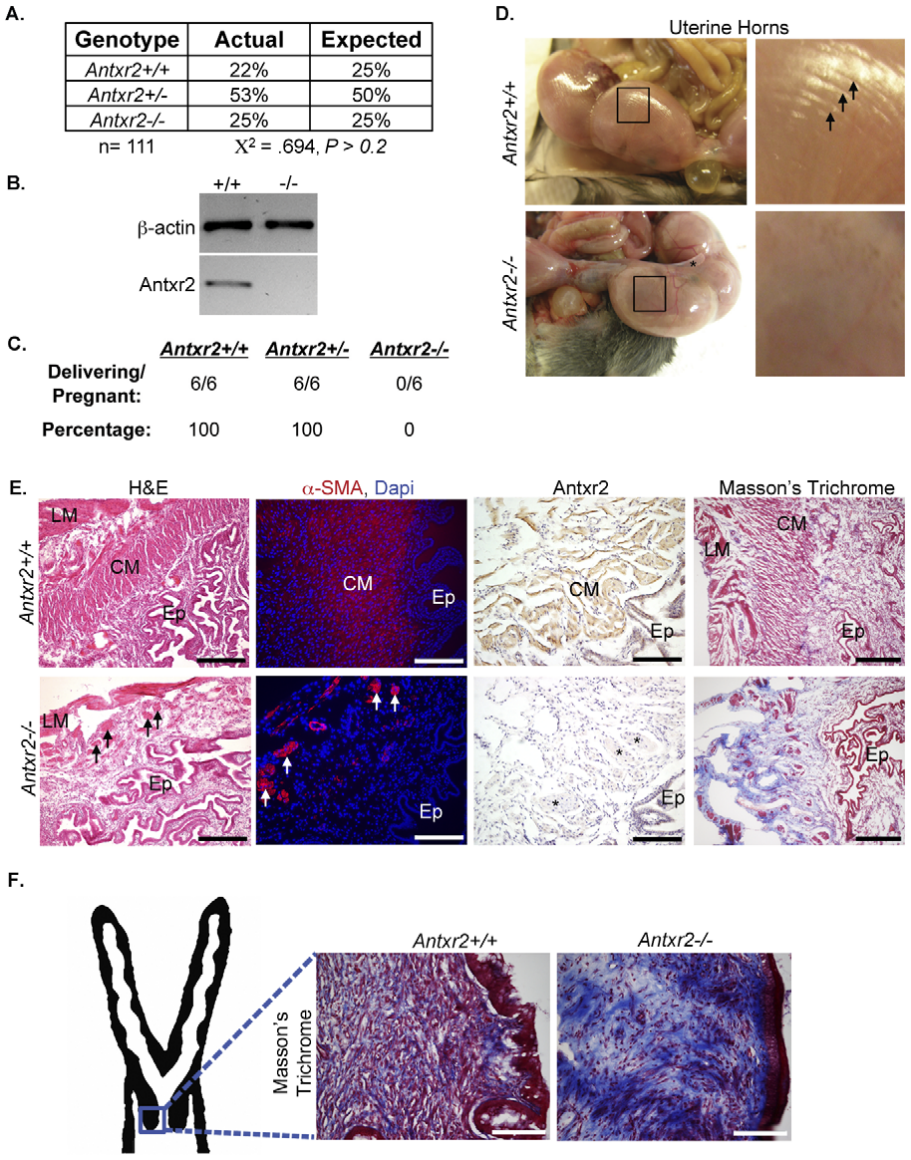
Antxr2 is required for murine parturition. (A) Genotyping of offspring from *Antxr2+/−* intercrosses revealed that *Antxr2−/−* mice are viable. (B) RT-PCR analysis of Antxr2 transcript expression in *Antxr2+/+* and *Antxr2−/−* MEFs. (C) Fertility analysis of 6-week-old female mice revealed that 100% of *Antxr2−/−* mice are unable to give birth. (D) Left panel- Analysis of uterine tissue isolated on GD18.5 showed that *Antxr2−/−* uterine horns lack muscle striations (black arrows in right panel) and exhibit poor uterine tone (asterisk). Right panel- blow ups of boxed areas in left panel to highlight lack of striations in *Antxr2−/−* uterine horns. (E) H&E staining and immunofluorescence for the smooth muscle cell marker, ∝-SMA, demonstrated that the circular and longitudinal myometrial cell layers are disrupted in GD18.5 *Antxr2−/−* uterine tissue (Ep, endometrial epithelium; LM, longitudinal myometrium; CM, circular myometrium). Arrows point to remaining bundles of myometrial cells in *Antxr2−/−* uterus. Immunostaining for Antxr2 revealed that the protein is expressed on myometrial cells in *Antxr2+/+* tissue (brown stain). Asterisks indicate bundles of myometrial cells that are negative for Antxr2 expression in *Antxr2−/−* tissue. Masson's Trichrome staining demonstrated high collagen content (blue color) in area of *Antxr2−/−* uterus where myometrial cell layers are usually present. (F) Masson's Trichrome staining of GD18.5 cervical tissue revealed a dense collagen network (blue color) in *Antxr2−/−* cervices. Scale bars, 150 µm.

To ascertain the function of Antxr2, we generated a conditional *Antxr2* knockout mouse. Exon 1 of *Antxr2* encodes the first 50 amino acids of the Antxr2 protein including a 26 amino acid signal peptide and initiating methionine. Thus, we targeted exon 1 for deletion using a *triloxP* targeting approach (Figure S1A). Deletion of exon 1 was accomplished by mating *triloxP* targeted male mice with female *Ella-Cre* transgenic mice. The maternally derived *Cre* is more efficient at producing total germline excision of the *loxP-*flanked exon 1 and NEO cassette (see Figure S1A) due to the presence of *Cre* in the oocyte. The *Antxr2−/−* mice described herein were on a mixed 129XC57BL/6 background. Intercrosses of *Antxr2+/−* mice produced progeny in the expected Mendelian ratios: 22%+/+, 53%+/−, 25%−/− of 111 offspring analyzed (Figure 1A), demonstrating that loss of *Antxr2* did not result in embryonic lethality. *Antxr2−/−* mice were viable at birth and developed normally, showing no striking phenotypic difference when compared with their wild type and heterozygous littermates at the macroscopic level. Histological analysis of skin, heart, lung, spleen, kidney, liver, intestine and bone did not reveal differences in organ development or organization at 1 month of age (data not shown). RT-PCR analysis on total RNA isolated from mouse embryonic fibroblasts (MEFs) confirmed that deletion of exon 1 led to a corresponding loss of *Antxr2* mRNA (Figure 1B).

To evaluate fertility of *Antxr2−/−* mice, we established timed matings. Young *Antxr2−/−* males were normal in their reproductive ability in that copulation plugs were detected and they impregnated female mice. 6-week-old *Antxr2−/−* females were also fertile. Once pregnant, *Antxr2−/−* females increased in body weight, but all of the mutant mice failed to deliver pups on the expected due date (gestational day 19) and died approximately one week later (Figure 1C). Necropsies revealed that the pups had died *in utero* and were beginning to degenerate. To determine if the parturition failure resulted from embryos dying during gestation, we analyzed embryo viability late in gestation. *Antxr2−/−* intercrosses (n = 7) and *Antxr2+/+* (n = 3) intercrosses were performed and we isolated embryos on gestational day 18.5 (GD 18.5), twelve hours before they were to be born. Regardless of genotype, all embryos were found to be alive on GD18.5, as determined by embryonic movement prior to dissection. No abnormalities were observed in either the size or gross morphology of the *Antxr2−/−* embryos or the associated placentas when compared to *Antxr2+/+* and *Antxr2+/−* embryos (data not shown). Furthermore, expression of embryonic Antxr2 did not result in the timely induction of parturition in *Antxr2−/−* dams. We established timed matings between *Antxr2−/−* females (n = 3) and *Antxr2+/+* males or *Antxr2+/+* females (n = 3) and *Antxr2−/−* males to generate *Antxr2+/−* embryos. Analysis of gestational length demonstrated that *Antxr2+/+* females carrying *Antxr2+/−* embryos gave birth on GD 19 whereas *Antxr2−/−* females carrying *Antxr2+/−* embryos consistently failed to give birth. These analyses suggested that abnormal progression of labor in the mother was the mechanism of death for the pregnant *Antxr2*−/− mice.

Parturition, the process of giving birth, requires the coordinated regulation of multiple signaling pathways in the ovary, uterus and cervix. In mice, pregnancy is maintained by continued synthesis of progesterone in the corpus luteum from fetal or maternal steroid percursors. At term, progesterone synthesis decreases and catabolism increases, producing a fall in serum progesterone, a process termed luteolysis [13]. Histological analysis of ovaries collected from *Antxr2+/+* and *Antxr2−/−* mice on GD 18.5 revealed the normal formation of corpus lutei with no overt structural abnormalities (Figure S1C). ELISA of serum from *Antxr+/+* and *Antxr2−/−* mice on GD 15.5 and 18.5 revealed that progesterone levels declined in both *Antxr2+/+* and *Antxr2−/−* mice as the pregnancies progressed to term (Figure S1D).

Parturition requires the onset of rhythmic contractions in the uterus and ripening/dilation of the cervix to allow for delivery of the embryo through the birth canal. The failure of either cervical ripening or adequate uterine contractions causes unsuccessful parturition [14]. To determine if either of these essential processes is disrupted in the *Antxr2−/−* mice, we isolated reproductive tracts on GD18.5 and conducted histological analysis of both the uterus and cervix. Gross inspection of reproductive tracts revealed that *Antxr2−/−* uterine tissue exhibited poor uterine tone (asterisk in Figure 1D) and lacked muscle striations. In contrast, the *Antxr2+/+* uterus was tightly wrapped around each embryo and exhibited visible muscle striations (arrows in Figure 1D). H&E staining demonstrated *Antxr2−/−* uteri lacked both circular (CM) and longitudinal (LM) myometrial cell layers, which was confirmed by alpha-smooth muscle actin (∝-SMA) immunostaining (Figure 1E). Immunostaining also demonstrated that Antxr2 is highly expressed in the uterine myometrium and confirmed lack of expression in the *Antxr2−/−* tissue (Figure 1E). To assess collagen content in the pregnant uterine tissue, we performed Masson's Trichrome staining and found increased fibrillar collagen deposition in the *Antxr2−/−* tissue in the area of the uterus where the longitudinal and circular myometrial cell layers normally reside (Figure 1E).

Normal cervical ripening is characterized by reorganization of the ECM [15]. To assess cervical ripening, GD18.5 cervical collagen content and organization was evaluated by staining with Masson's Trichrome. In *Antxr2+/+* cervices, we observed a loose array of collagen fibers, an indicator of compliant tissue. *Antxr2−/−* cervices exhibited dense, compact, heavily stained collagen fibrils, which are characteristic of inelastic tissue (Figure 1F). Taken together, the disorganized nature of the myometrium in the uterus and the dense collagen network in the cervix suggests that the parturition defect in the *Antxr2−/−* mice is due to inadequate uterine contractions and a failure in cervical ripening.

### Nulliparous Aged *Antxr2*−/− Mice Develop Severe Fibrosis in the Uterus and Cervix

**Figure 2:**
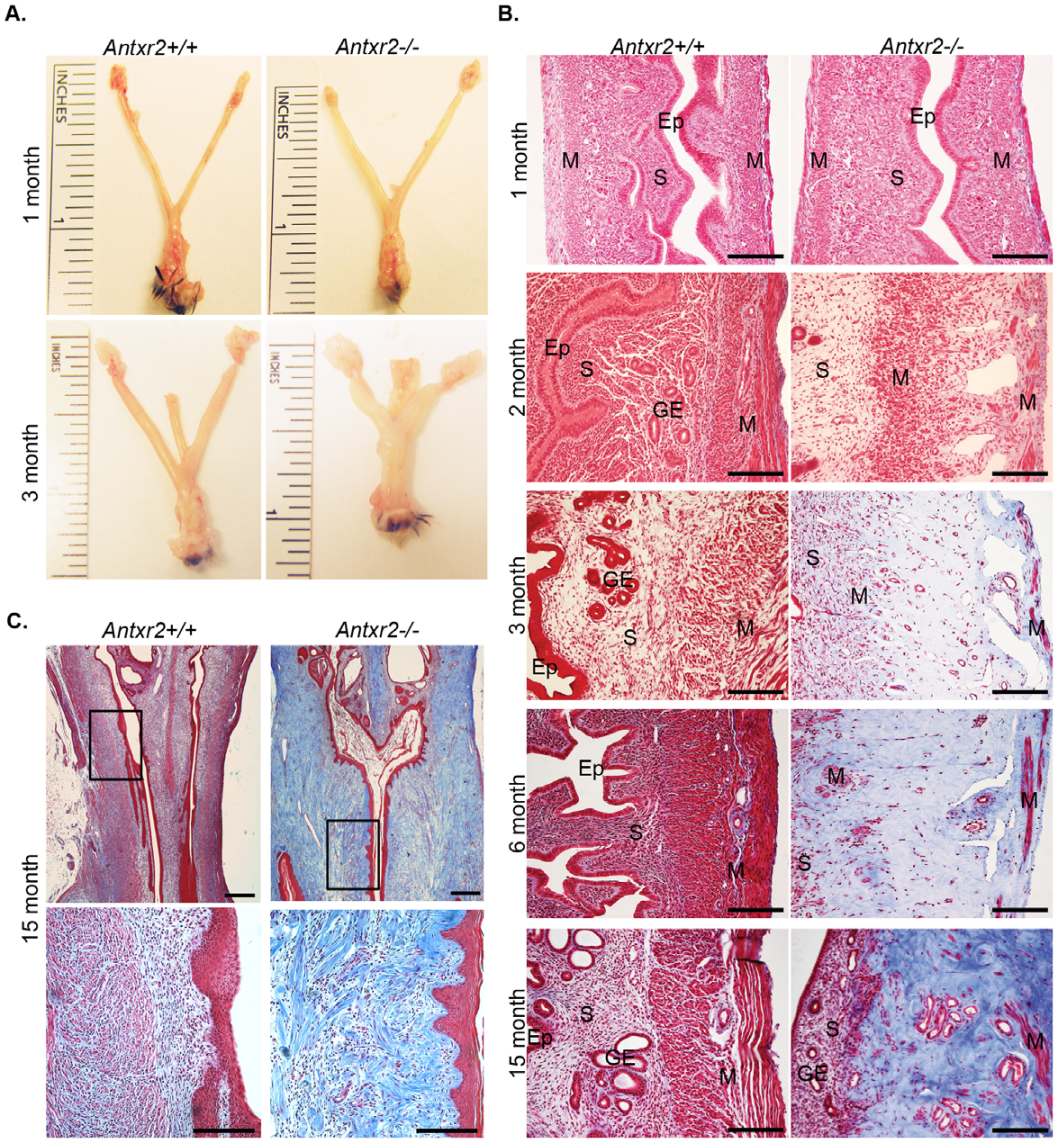
Reproductive tracts isolated from aged nulliparous *Antxr2−/−* female mice exhibit an altered morphology with severe fibrosis. (A) Comparison of reproductive tracts isolated from *Antxr2+/+* and *Antxr2−/−* mice at one month and three months of age. Sexually mature, (three-month-old) *Antxr2−/−* uteri are shortened and thickened compared to *Antxr2+/+*. Tissue lying between the uterine horns in the three-month images is the colon. (B) Masson's Trichrome staining of reproductive tracts demonstrated that there is progressive collagen fibrosis (blue color) in the uterus as *Antxr2−/−* mice age. Ep, endometrial epithelium; S, stroma; M, myometrium; GE, glandular epithelium. Scale bars, 150 µm. (C) Masson's Trichrome staining of fifteen-month-old cervical tissue demonstrated that *Antxr2−/−* mice have cervices with increased collagen content (blue color). Bottom panel is boxed image at higher magnification. Top panel scale bars, 400 µm. Bottom panel scale bars, 150 µm. Three mice of each genotype (*Antxr2+/+* and *Antxr2−/−*) were evaluated for each time point. Representative images for each time point are shown.

In addition to the parturition defect, we observed that older *Antxr2−/−* females, from 2 months of age and beyond, had problems with fertility. Mating young (6-week-old), sexually mature *Antxr2−/−* females produced pregnancies that were carried to term but resulted in defective parturition. In contrast, older *Antxr2−/−* females, aged 2 to 6-months, had difficulty carrying a pregnancy to term. Fertility analysis revealed that these *Antxr2−/−* females were able to get pregnant as evidenced by plug formation and subsequent weight gain, however, approximately half of the pregnant animals miscarried their litters. Fertility analysis of female mice aged 7-months-old and beyond revealed that they were unable to get pregnant. Consequently, we isolated reproductive tracts from both young and aged nulliparous *Antxr2−/−* mice for analysis. Reproductive tracts isolated from one-month-old prepubescent mice looked similar in overall appearance (Figure 2A top panel), but reproductive tracts isolated from sexually mature 3-month-old mice displayed striking differences in morphology (Figure 2A bottom panel). The *Antxr2−/−* reproductive tracts had a shortened, thickened shape in comparison to the thin, elongated reproductive tracts from *Antxr2+/+* animals. This phenotype was observed for every nulliparous *Antxr2−/−* female mouse we evaluated (n = 18, aged 3–15 months). Masson's trichrome staining did not reveal overt structural abnormalities or changes in collagen deposition in prepubescent *Antxr2−/−* uteri (Figure 2B, one month panel). However, sexually mature *Antxr2−/−* uteri were characterized by collagen fibrosis (Figure 2B, panels 2, 3, 6, 15 month). The fibrosis progressed as the mice aged, resulting in a thickened, collagen dense, acellular stroma and the disappearance of normal uterine architecture (Figure 2B). Similarly, cervical tissue isolated from 15-month-old *Antxr2−/−* mice exhibited increased collagen content (Figure 2C) as compared to *Antxr2+/+* tissue. We also examined collagen deposition in the ovaries of aged mice. Unlike what had been reported for *Antxr1−/−* mice (9), we did not observe increased collagen content in ovaries isolated from either 3-month-old or 6-month-old *Antxr2−/−* mice. *Antxr2−/−* ovaries appeared normal with the presence of follicles in various stages of maturation (Figure S2). We conclude that the extensive fibrosis throughout the reproductive tract in aged *Antxr2−/−* mice impairs fertility.

### The Myometrium is Disrupted in Nulliparous Aged *Antxr2*−/− Mice

**Figure 3:**
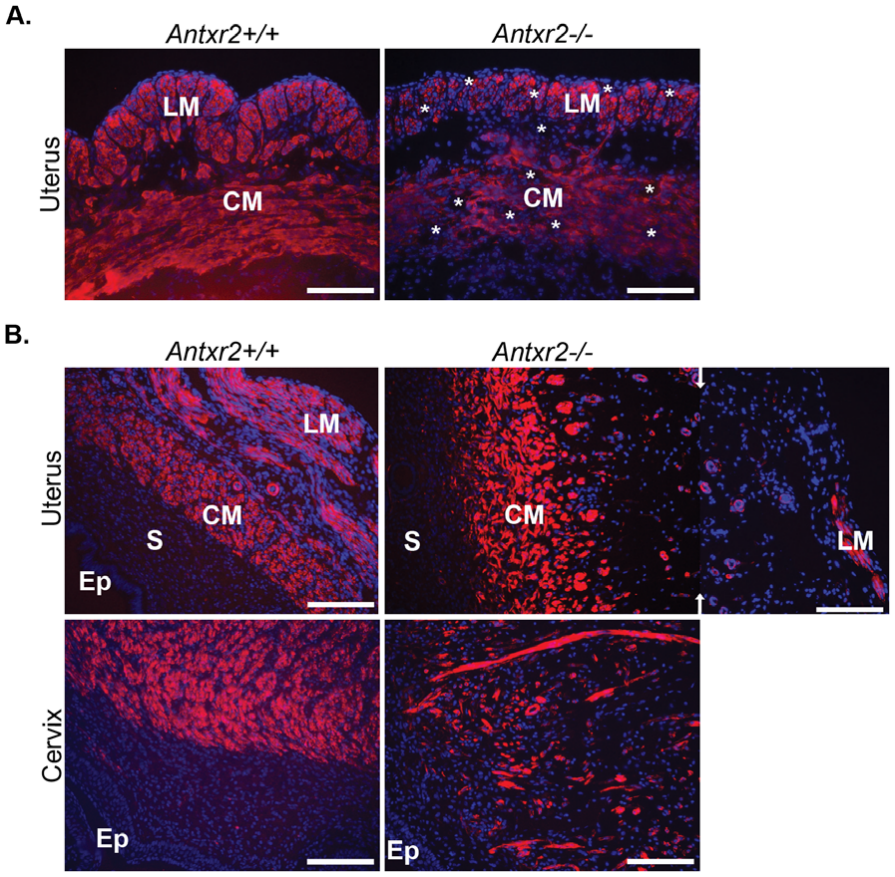
The myometrium is disrupted in aged nulliparous *Antxr2−/−* reproductive tracts. (A) Immunofluorescence for ∝-SMA (red) demonstrated well-defined circular and longitudinal myometrial cell layers that were beginning to disassociate in 6.5 week *Antxr2−/−* tissue. Space between myometrial cell bundles is indicated by asterisks in the *Antxr2−/−* tissue. (B) ∝-SMA (red) staining of three-month-old tissue demonstrated that the disassociation between the myometrial layers had progressed in *Antxr2−/−* uterine and cervical tissue. In the uterus, the dispersal was such that the remaining muscle bundles could not be captured together in the same photographic frame. Arrows indicate where two images were placed together in order to demonstrate the distance between the longitudinal and circular myometiral cell layers in the *Antxr2−/−* uterus. DAPI (blue color) is used for nuclear staining. Scale bars, 150 µm.

The normal architecture of the uterine wall consists of an inner circular layer of myometrial (CM) cells, an intervening vascular space and an outer longitudinal layer of myometrial (LM) cells as seen in *Antxr2+/+* mice (Figure 3). Immunofluorescent staining with ∝-SMA revealed well-defined, tightly packed CM and LM layers. Uteri isolated from nulliparous *Antxr2−/−* mice presented disorganized CM and LM layers, similar to that seen in uteri from pregnant *Antxr2−/−* mice. As early as 6.5 weeks of age, the CM and LM layers were beginning to loosen resulting in increased intercellular space between bundles of muscle cells (see asterisks in Figure 3A). This loosening progressed as the mice aged. The CM in uteri isolated from 3-month-old *Antxr2−/−* mice consisted of a poorly defined layer of scattered smooth muscle cells. The space between the CM and LM layers had become greatly distended. We represent this in Figure 3B where two pictures of *Antxr2−/−* uterine morphology are placed together in order to capture the same area represented in one picture of *Antxr2+/+* tissue. The LM was almost completely ablated in the *Antxr2−/−* tissue with only a few muscle cell bundles at the periphery of the uterus (Figure 3B, merged top panel). We observed a similar smooth muscle cell phenotype in the cervix (Figure 3B, bottom panel). TUNEL staining did not reveal an increase in myometrial cell death in the *Antxr2−/−* tissues analyzed indicating that loss of cells due to apoptosis is gradual over months or not a mechanism of muscle cell loss. However, we did detect increased cell death in luminal and glandular epithelial cells in uterine tissue aged 6 months and beyond (data not shown). These results demonstrate that in both pregnant (Figure 1E) and non-pregnant (Figure 3) states, Antxr2 has a critical role in the maturation or maintenance of the myometrium. It is also interesting to note that in the uteri of both pregnant and aged nulliparous *Antxr2−/−* mice, the loss of myometrial cells is associated with ECM protein accumulation. The myometrium has been demonstrated to produce MMP2 during postpartum involution of the rat uterus [16]. Taking this into account, our data suggests that the myometrium is also important for matrix remodeling in the cycling uterus and during pregnancy.

### Vascular Changes and Inflammation Accompany Fibrosis in the Nulliparous *Antxr2*−/− Reproductive Tract

**Figure 4:**
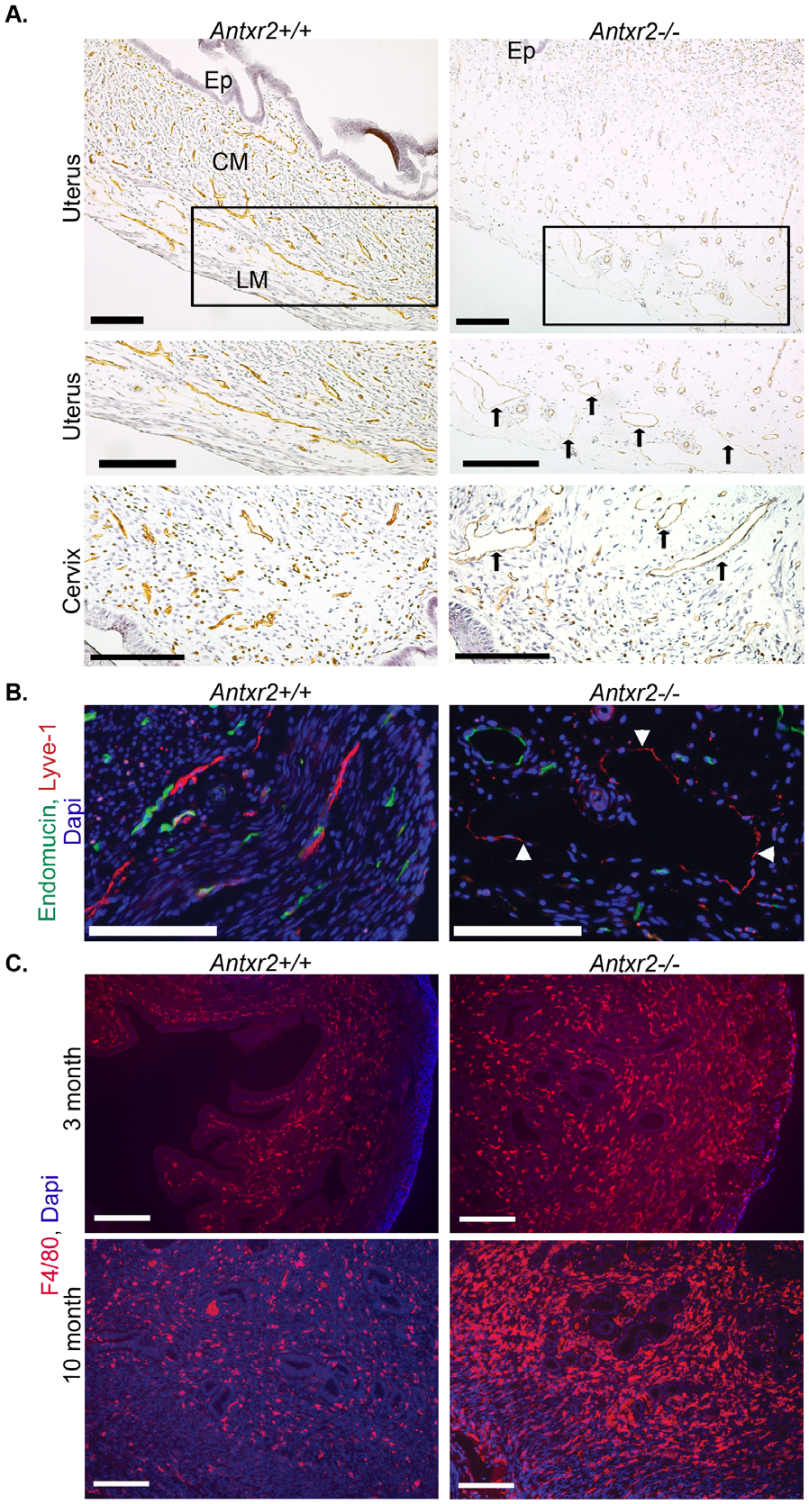
Uterine fibrosis in aged nulliparous *Antxr2−/−* mice is accompanied by atypical vasculature and inflammation. (A) CD31 immunostaining (brown color) of three-month-old reproductive tracts reveal atypical/open blood vessels (arrows) throughout the *Antxr2−/−* uterus and cervix. Boxed areas are blown up to highlight vasculature. Ep, endometrial epithelium; CM, circular myometrium; LM, longitudinal myometrium. Scale bars on uterus photos, 200 µm. Scale bars on cervix photos, 150 µm. (B) Coimmunofluoresence for blood endothelial cell marker, endomucin (green color), and lymphatic endothelial cell marker, lyve-1 (red color) on three-month-old uterine tissue. DAPI (blue color) is used for nuclear staining. Lymphatic vessels (arrowheads) in *Antxr2−/−* tissue are enlarged. Scale bar, 100 µm. (C) Immunofluorescent staining for macrophage marker, F4/80 (red color), revealed an increased inflammatory response in three-month-old and ten-month-old *Antxr2−/−* uterine tissue. DAPI (blue color) is used for nuclear staining. Scale bars, 200 µm.

Staining for the endothelial marker, CD31, revealed atypical vessels in the uterus and the cervix of *Antxr2−/−* mice when compared to that of *Antxr2+/+* vessels (Figure 4A, arrows). When uterine tissue was sectioned in the same orientation, vessels in the *Antxr2+/+* tissue had collapsed lumens while vessels in the *Antxr2−/−* tissue had open lumens. CD31 staining in the *Antxr2−/−* tissue was also more faint. We have detected a reduction in CD31 at the cell surface when performing flow cytometry on human umbilical venous endothelial cells (HUVEC) with ANTXR2 knocked down via RNA interference (RNAi) (data not shown) and there may be reduced CD31 expression on the endothelium in *Antxr2−/−* tissue.

As CD31 does not differentiate between blood vasculature and lymphatic vasculature, we also performed coimmunofluorescence using the blood endothelial cell marker, endomucin, and the lymphatic endothelial cell marker, lyve-1. In *Antxr2+/+* tissue, lymphatic vessels were collapsed and resided within the CM and LM layers (Figure 4B). In the *Antxr2−/−* tissue, co-staining demonstrated that the lymphatic vessels were grossly dilated (Figure 4B, white arrowheads).

In addition to changes in the blood and lymphatic vasculature, there was a far greater infiltration of inflammatory cells, detected as F4/80 positive macrophages (Figure 4C). There is a resident population of macrophages in the uterus [17], however, if ECM accumulation in the *Antxr2−/−* reproductive tract is likened to a wound, it is possible that dilation of blood and lymphatic vessels allows for influx of macrophages into the tissue in order to facilitate tissue repair. These histopathological changes are hallmarks of fibrotic tissue that we believe are secondary to the uterine fibrosis rather than the result of losing Antxr2 expression in blood endothelium, lymphatic endothelium or macrophages. In support of this hypothesis, we have generated mice with deletion of *Antxr2* in the blood endothelium using a VE-cadherin *Cre* driver line. Reproductive tracts from female VE-Cadherin *Cre;Antxr2^fl/fl^* mice do not have ECM accumulation nor do they have atypical/open blood vessels (data not shown).

### Uterine Fibrosis in Nulliparous Aged *Antxr2*−/− Mice is Characterized by Increased Collagen and Fibronectin Content

**Figure 5:**
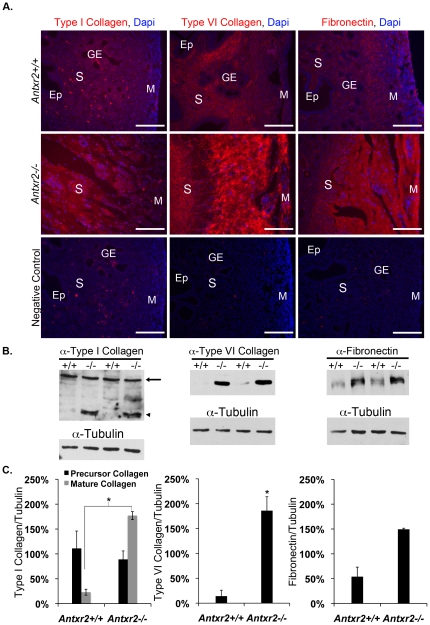
Increased collagen and fibronectin content in aged nulliparous *Antxr2−/−* uterine tissue. (A) Immunofluorescent staining of uterine tissue isolated from six-month-old mice demonstrated increased type I collagen, type IV collagen and fibronectin deposition in the *Antxr2−/−* tissue. DAPI (blue) was used for nuclear staining. Negative controls demonstrate specificity of the antibodies. Ep, endometrial epithelium; S, stroma; M, myometrium; GE, glandular epithelium. Scale bars, 150 µm. (B) Uterine lysates from six-month-old mice, *Antxr2+/+* (n = 2) and *Antxr2−/−* (n = 2), were immunoblotted for type I collagen (precursor type I collagen indicated by arrow and mature type I collagen indicated by arrowhead), type VI collagen and fibronectin. Alpha tubulin is shown as a loading control. (C) Densitometric analysis of blots in panel B presented as relative levels of designated ECM protein normalized to respective alpha tubulin. The mean ± the standard deviation are represented, * = *P*<0.05.

We assessed the types and amounts of fibrillar collagens or other ECM proteins present in uterine tissue, focusing our analysis on predicted ECM ligands for ANTXRs. Immunostaining revealed that type I collagen, type VI collagen and fibronectin content is increased in uteri isolated from 6-month-old *Antxr2−/−* mice compared to that of *Antxr2+/+* (Figure 5A). In order to quantify the changes in ECM content, uterine lysates from 6-month-old mice were immunoblotted for type I collagen, type VI collagen, fibronectin, and tubulin as a loading control (Figure 5B) and densitometery was used to quantify protein bands. We found that there was no significant change in the amount of precursor type I collagen present in *Antxr2−/−* uterine tissue (arrow in Figure 5B), however, there was a significant 7 fold increase in the amount of mature type I collagen in *Antxr2−/−* uteri as compared to *Antxr2+/+* (*P*<.005) (Figure 5C). Similarly, the amount of type VI collagen present in *Antxr2−/−* uteri was 13 times that of *Antxr2+/+* uteri (*P*<.05) (Figure 5C). There was also a trend towards increased fibronectin content in the *Antxr2−/−* uteri, however it did not reach significance (*P* = .08) when compared to *Antxr2+/+* levels (Figure 5C). Immunostaining revealed that accumulation of these same ECM proteins was more pronounced in 10-month-old *Antxr2−/−* tissue (Figure S3). The uterus is a dynamic organ that undergoes extensive ECM remodeling with each round of the estrus cycle [18]. The accumulation of uterine ECM proteins (Figures 2, 5 and S3) as *Antxr2−/−* mice age suggests a defect in the remodeling process.

### Matrix Metalloproteinase 2 Activity is Impaired in Cells and Tissue Deficient for *Antxr2*

The uterine endometrium and associated stroma undergoes extensive remodeling during post-pubertal life in response to the estrus cycle [19]. Part of this remodeling process involves the synthesis and degradation of ECM components, especially interstitial collagens and basement membranes [19]. Matrix metalloproteinases (MMPs) are the prime mediators of ECM protein degradation and their expression is differentially regulated throughout the estrus cycle in the uterus [19]. The gradual accumulation of multiple ECM components in *Antxr2*−/− uteri led us to hypothesize that there was a defect in a factor(s) known to degrade multiple and diverse ECM proteins.

**Figure 6:**
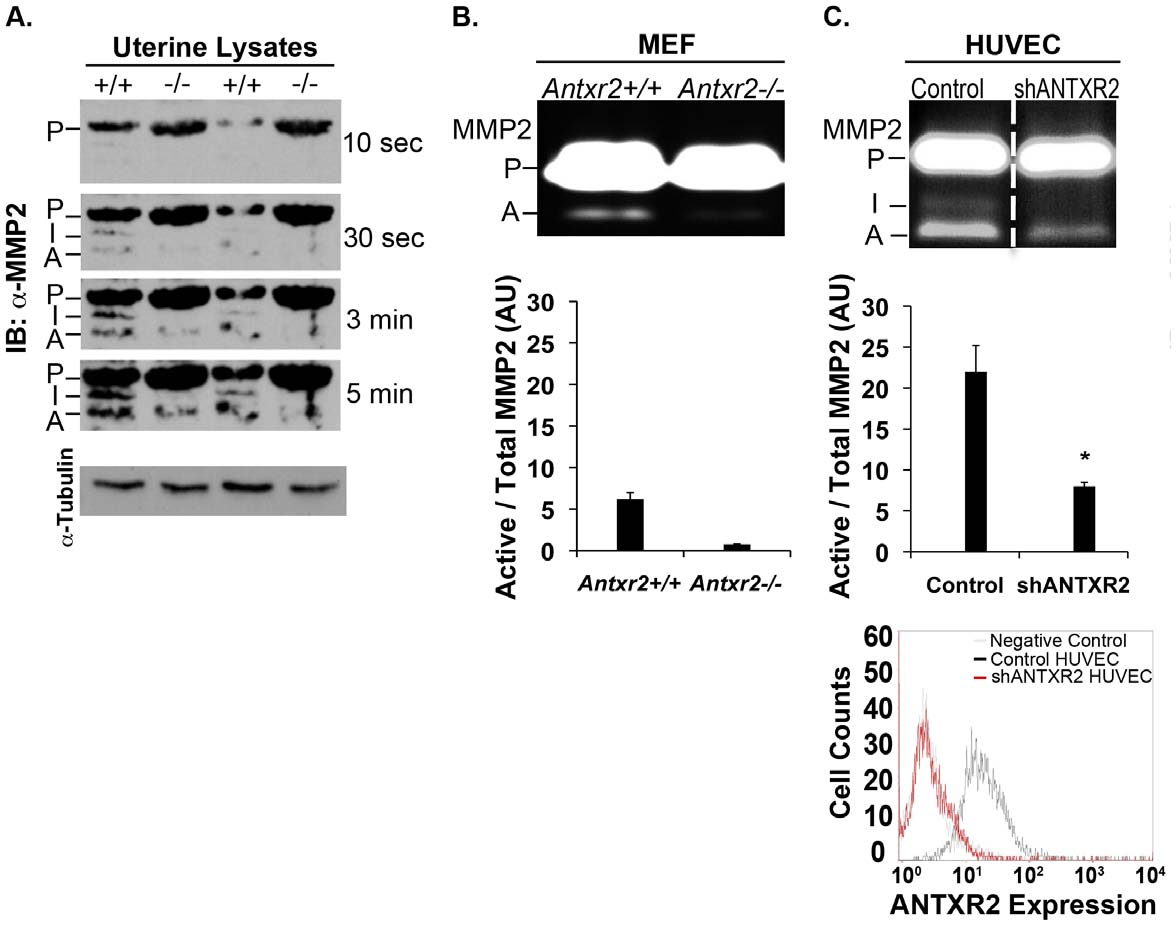
Reduced MMP2 activity in Antxr2 deficient tissue and cells. (A) Uterine lysates from six-month-old mice, *Antxr2+/+* (n = 2) and *Antxr2−/−* (n = 2), were immunoblotted for MMP2 and demonstrated that there are reduced levels of active MMP2 in the *Antxr2−/−* tissue (P = the pro form of MMP2, I = the intermediate form of MMP2 and A = the active form of MMP2). Alpha tubulin was used as a loading control. (B) Gelatin zymography revealed reduced levels of active MMP2 in conditioned medium from *Antxr2−/−* MEFs. A representative of two independent experiments is shown. For each experiment, samples were run in duplicate. The graph below the zymogram gel represents the relative levels of active to total MMP-2 (pro+intermediate+active) as quantified by densitometry and shows the mean ± standard deviation (*P* = .06). (C) Conditioned medium from HUVEC cell lines with knock down of ANTXR2 expression (shANTXR2 HUVEC) had reduced MMP2 activity as determined by gelatin zymography. The vertical dotted line reflects the fact that different parts of the same gel were placed next to each other in the figure for ease of comparison. A representative of two independent experiments is shown. For each experiment, the samples were run in quadruplicate. The graph below the zymogram gel represents the relative levels of active to total MMP-2 (pro+intermediate+active) as quantified by densitometry and shows the mean ± standard deviation (*P*<.05). The bottom panel is a histogram from flow cytometry analysis of retrovirally-infected HUVEC scrambled shRNA (control) or ANTXR2 shRNA (shANTXR2) cell lines. The histogram shows decreased ANTXR2 expression at the cell surface of the shANTXR2 HUVEC line.

To evaluate MMP status *in vivo*, we used uterine lysates from 6-month-old *Antxr2+/+* (n = 2) and *Antxr2−/−* (n = 2) mice and performed western blotting. A short exposure of the film (10 seconds) revealed increased level of proMMP2 in the *Antxr2−/−* tissue. In the *Antxr2+/+* uterine lysates, intermediate and active MMP2 protein was clearly detected in longer film exposures (30 seconds, three minutes and five minutes, Figure 6A). Despite equal loading of protein lysate, as evidenced by the tubulin loading control, active MMP2 was not readily detectable in the *Antxr2−/−* tissue until the three and five minute exposure times (Figure 6A). The intermediate form of MMP2 was not detected in the *Antxr2−/−* tissue. Thus, MMP2 processing is defective in the uteri of *Antxr2−/−* female mice.

We assessed MMP2 activity in *Antxr2+/+* and *Antxr2−/−* mouse embryonic fibroblasts (MEFs). Gelatin zymography revealed that there were reduced levels of active MMP2 in conditioned medium from *Antxr2−/−* MEFs (Figure 6B). When quantified using densitometery, the ratio of active MMP2 to total MMP2 was eight fold higher in *Antxr2+/+* MEFs when compared to *Antxr2−/−* MEFs (Figure 6B). This difference was almost statistically significant (*P* = .06). Without artificial activation by organomercurials, it is very difficult to detect endogenous activation of MMP2 in MEFs. Therefore, we believe the lack of significance is due to the low level of active MMP2 detected from the *Antxr2+/+* cells.

We used RNAi to knockdown ANTXR2 in HUVEC, a cell type that requires ANTXR2 for endothelial proliferation and network formation, processes which could be affected by impaired MMP activity [6]. Flow cytometry was performed to detect knockdown of ANTXR2 by shRNA [6] at the cell surface (see histogram Figure 6C). Similar to the MMP defects seen in MEFs, gelatin zymography showed that MMP2 levels were reduced in knockdown lines compared to control HUVEC (Figure 6C). Quantification of the zymography bands demonstrated that the ratio of active to total MMP2 was 2.75 times higher in the control cells when compared to the knockdown cells (*P* = .003) (Figure 6C). Thus, two different cell types deficient for ANTXR2 expression had reduced MMP2 activation.

### Anthrax Toxin Receptor 2 Regulates Membrane Type I Matrix Metalloproteinase Activity

The classic model for activation of MMP2 is through the formation of a trimolecular complex comprised of MT1-MMP, TIMP-2 and pro MMP2 [20]. MT1-MMP interacts via its N-terminal domain with the N terminus of TIMP-2 and this complex forms a receptor for pro MMP2. Pro MMP2 bound to this receptor is initially cleaved to its intermediate form by an adjacent active MT1-MMP. The second stage of MMP2 processing results in a fully active form and involves an autocatalytic event that requires an active MMP2 protein acting in *trans* [21], [22], [23]. Taking this mechanism of MMP2 activation into account, the increase in pro MMP2, the reduction in active MMP2 and the fact that the intermediate form of MMP2 was not detected in *Antxr2−/−* uterine tissue suggested that Antxr2 might be affecting MT1-MMP function.

**Figure 7:**
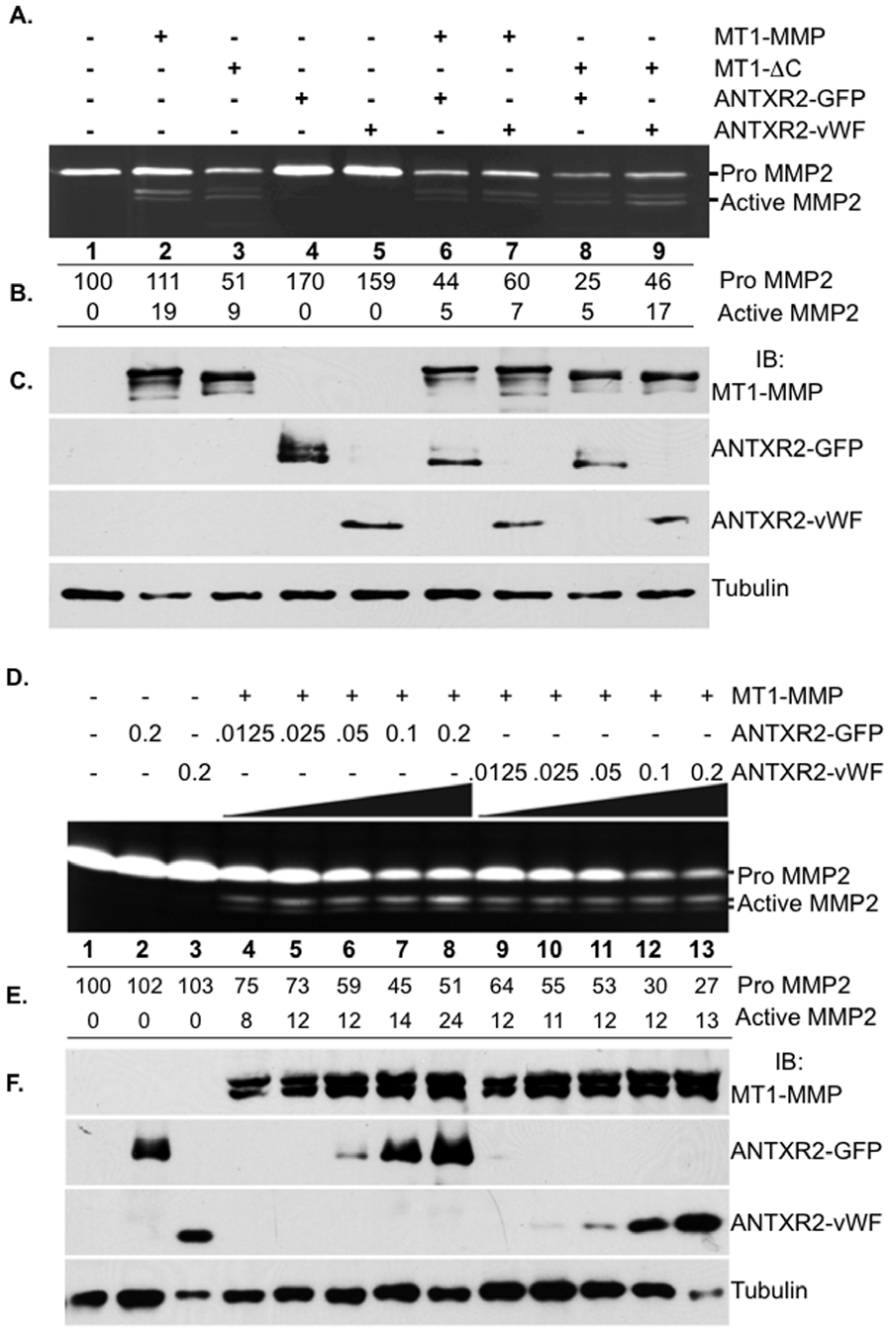
ANTXR2 positively regulate MT1-MMP activity. (A) Zymographic analysis of conditioned medium from 293T cells transfected with empty vector (lane 1), MT1-MMP (lane2), MT1-ΔC (lane 3), ANTXR2-GFP (lane 4), ANTXR2-vWF (lane 5), MT1-MMP and ANTXR2-GFP (lane 6), MT1-MMP and ANTXR2-vWF (lane 7), MT1-ΔC and ANTXR2-GFP (lane 8), or MT1-ΔC and ANTXR2-vWF (lane 9) revealed that co-expression of either MT1-MMP or MT1-ΔC and ANTXR2-GFP or ANTXR2-vWF led to enhanced pro MMP2 activation over expression of either MT1-MMP or MT1-ΔC alone. (B) Table under the zymogram in panel A represents densitometric quantification of the pro and active MMP2 bands. Numbers are in percentile of relative intensity in relation to the empty vector control, lane 1. (C) Immunoblots for MT1-MMP, ANTXR2-GFP, ANTXR2-vWF and Tubulin from the 293T cell lysates corresponding to the zymography experiment in panel A. (D) Zymographic analysis of conditioned medium from 293T cells co-expressing MT1-MMP and varying concentrations of ANTXR2-GFP or ANTXR2-vWF revealed that MT1-MMP activity is dependent on ANTXR2 expression levels. (E) Table under the zymogram represents densitometric quantification of the pro and active MMP2 bands. Numbers are in percentile of relative intensity in relation to the empty vector control, lane 1. (F) Immunoblots for MT1-MMP, ANTXR2-GFP, ANTXR2-vWF and Tubulin from the 293T cell lysates corresponding to the zymography experiment in panel D. For each zymogram panel, a representative of two independent experiments is shown.

To address this hypothesis, 293T cells were transfected with either wild type MT1-MMP or a catalytically active variant of MT1-MMP (MT1-ΔC), along with either full length ANTXR2 with a GFP tag at the carboxy terminus (ANTXR2-GFP) or a truncated variant of ANTXR2 consisting of the vWF domain (ANTXR2-vWF). Cell surface MT1-MMP activity was measured as the ability of cells to activate pro MMP2, a known substrate of MT1-MMP, and was evaluated using gelatin zymography. In our system, we defined enhanced MT1-MMP activation as a reduction in the amount of pro MMP2 detected. A corresponding increase in the amount of active MMP2 is more difficult to detect, as the half-life of the activated MMP2 enzyme is very short due to autocatalysis. Tables (Figure 7B and 7E) under the zymogram gels indicate densitometric quantification of the pro and active MMP2 bands and numbers are expressed as the percentiles of relative intensity in relation to the pro MMP2 band in the empty vector control (lane 1).

Expression of MT1-MMP in 293T cells showed trace levels of activated MMP2 (Figure 7A, lane 2) and catalytically active MT1-ΔC showed enhanced pro MMP2 activation over wild type MT1-MMP in the conditioned medium (Figure 7A, lane 3). Expression of either ANTXR2-GFP or ANTXR2-vWF alone had no affect on pro MMP2 processing (Figure 7A, lanes 4 & 5). Co-expression of MT1-MMP and either ANTXR2-GFP or ANTXR2-vWF consistently showed greater MMP2 activation than cells expressing MT1-MMP alone (Figure 7A, compare lane 2 to lanes 6 & 7). The processing of pro MMP2 was further enhanced in cells co-expressing MT1-ΔC and either ANTXR2-GFP or ANTXR2-vWF (Figure 7A, lanes 8 & 9). Immunoblotting confirmed that the 293T cells were expressing MT1-MMP, MT1-ΔC, ANTXR2-GFP and ANTXR2-vWF and the appropriate combinations thereof (Figure 7C). We obtained similar results when 293T cells co-expressed MT1-MMP and the ANTXR2 homolog, ANTXR1 (Figure S4). Co-expression of MT1-MMP and either ANTXR1-GFP or ANTXR1-vWF consistently showed pro MMP2 activation levels comparable to that achieved by co-expression of MT1-MMP and ANTXR2 (Figure S4). This data demonstrates that ANTXR1 and ANTXR2 positively regulate MT1-MMP activity. Furthermore, the vWF domain, present on the extracellular side of the ANTXR proteins, is sufficient for promoting this activity.

To provide additional evidence in support of a role for ANTXR2 as a regulator of MT1-MMP activity, we analyzed MT1-MMP activity in response to various doses of ANTXR2-GFP or ANTXR2-vWF. We found that increased expression of ANTXR2-GFP resulted in a dose dependent decrease in pro MMP2 levels (Figure 7D, lanes 4–8). We also detected a corresponding increase in active MMP2 levels. Densitometric quantification of the pro and active MMP2 bands confirmed the dose response (Figure 7E). Co-expression of MT1-MMP with increasing amounts of ANTXR2-vWF also resulted in a dose dependent decrease in pro MMP2 levels (Figure 7D, lanes 9–13), however, we were unable to capture a corresponding increase in active MMP2 levels. As mentioned earlier, this may be due to the short half-life of the active enzyme. Alternatively, it may suggest that the ANTXR2-vWF variant has partial function. Immunoblotting confirmed that the 293T cells were expressing MT1-MMP and increasing amounts of ANTXR2-GFP and ANTXR2-vWF (Figure 7C). The dose dependent response was also evident upon evaluation of MT1-ΔC activity (Figure S4B, ANTXR2-GFP lanes 4–8, ANTXR2-vWF lanes 9–13). Thus, MT1-MMP processing of pro MMP2 is dependent on the ANTXR2 expression levels in cells.

### Anthrax Toxin Receptor 2 and Membrane Type I Matrix Metalloproteinase Interact

**Figure 8:**
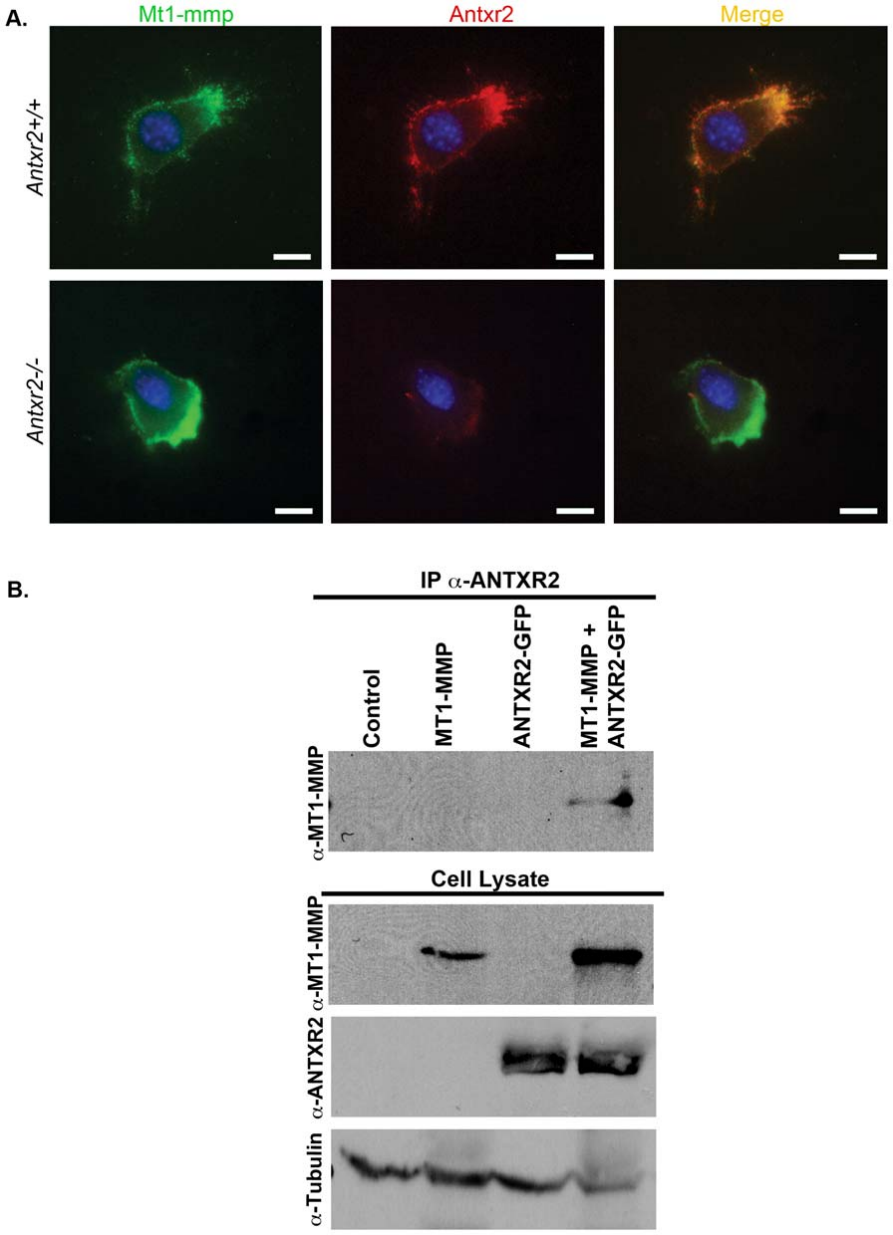
ANTXR2 and MT1-MMP colocalize and are found in complex. (A) Coimmunofluorescence for Mt1-mmp (green color) and Antxr2 (red color) on *Antxr2+/+* and *Antxr2−/−* MEFs demonstrate that MT1-MMP and ANTXR2 colocalize at the cell surface (orange). DAPI (blue color) is used for nuclear staining. Scale bars, 5 µm. (B) 293T cells were transfected with empty vector, MT1-MMP, ANTXR2-GFP or MT1-MMP and ANTXR2-GFP. Cell lysates were immunoprecipitated with antibody against ANTXR2 followed by western blotting to detect MT1-MMP. The coimmunoprecipitation revealed that ANTXR2 and MT1-MMP are found together in complex. A representative of two independent experiments is shown.

We next asked if ANTXR2 and MT1-MMP interact in cells. To address this question, we studied expression and interaction of MT1-MMP and ANTXR2 in MEFs and in transfected 293T cells. Immunofluorescent double labeling of unpermeabilized MEFs demonstrated that Mt1-mmp protein was present in a punctate membranous staining pattern on the cell surface of both *Antxr2+/+* and *Antxr2−/−* MEFs (Figure 8A). In *Antxr2+/+* MEFs, Antxr2 localized to the cell surface and was found to colocalize with Mt1-mmp (Figure 8A). Antxr2 was not expressed in *Antxr2−/−* MEFs as expected (Figure 8A).

Coimmunoprecipitation experiments were carried out to confirm the association between the two proteins. 293T cells were transfected to express MT1-MMP, ANTXR2-GFP or MT1-MMP and ANTXR2-GFP and cell lysates were subjected to immunoprecipitation with an ANTXR2 antibody. The immunoprecipitated lysate was analyzed by western blotting with anti-MT1-MMP antibody. The experiment revealed that a 60 kDa protein representing MT1-MMP coimmunoprecipitated with ANTXR2 (Figure 8B), indicating that ANTXR2 can localize to a complex with MT1-MMP.

## Discussion

While there is a detailed understanding of ANTXR interaction with the tripartite anthrax toxin, physiological ANTXR activity has remained poorly defined. In order to evaluate endogenous function, we generated an *Antxr2* knockout mouse by deleting exon 1 of the *Antxr2* gene. *Antxr2−/−* mice were viable, however, we discovered that Antxr2 is required for parturition in young female mice and for preserving fertility in older female mice. Analysis of *Antxr2−/−* reproductive defects revealed that Antxr2 is required for myometrial cell viability and ECM homeostasis in the murine uterus and cervix and led us to discover a novel mechanism of action for ANTXR2 as a positive regulator of MT1-MMP activity. This finding has implications for how ECM levels are regulated in developing, regenerating and pathological tissues.

The reproductive defects in female *Antxr2−/−* mice varied depending on the age of the mice at time of analysis. Young female *Antxr2−/−* mice mated immediately after reaching sexual maturity at 6 weeks of age were fertile. They were easily impregnated, carried their litters to term, but exhibited a block in parturition, the process of giving birth. Coordinated uterine contractions and cervical ripening are two processes that are essential to the progression of labor. We discovered that both of these processes were defective in the *Antxr2−/−* mice. Histological evaluation of the pregnant *Antxr2−/−* uterus at the end of the gestational period revealed loss of the circular and longitudinal myometrial cell layers (Figure 1D & E). This loss most likely resulted in nonproductive uterine contractions. Additionally, the *Antxr2−/−* cervix was found to be collagen dense indicating defective ECM remodeling and by extension defective cervical ripening (Figure 1F).

Older sexually mature *Antxr2−/−* female mice, aged 2 to 6 months, demonstrated impaired fertility. Approximately half of the animals that were successfully impregnated would miscarry their litters. The other half carried their litters to term, but could not give birth, exhibiting parturition defects as described above. The underlying cause of impaired fertility in the older *Antxr2−/−* mice was likely due to defects in uterine receptivity as suggested by the atypical *Antxr2−/−* uterine morphology observed at the 2 to 6 month time points, which included mild fibrosis and disorganized myometrial muscle layers (Figure 2B). Future studies focusing on ECM remodeling at various stages of pregnancy such as decidualization and placentation in *Antxr2−/−* mice may shed light on the fertility defect, however, changes in hormone expression profiles and downstream signaling cascades should not be ruled out.

*Antxr2−/−* female mice aged 7 months and beyond were infertile. Mating these mice did not result in pregnancies. Analysis of ovaries from older *Antxr2−/−* mice did not reveal overt changes in ECM content that might interfere with follicular maturation or oocyte production and release. Therefore, it is logical to assume that the uterus is unable to support implantation due to the fact that the pronounced fibrosis in aged *Antxr2−/−*uterine tissue completely destroys normal uterine architecture (Figure 2B).

While we have documented various reproductive issues in *Antxr2−/−* female mice, *Liu et. al.* reported that female *Antxr2*−/− mice become pregnant but fail to support normal embryonic development, without further elaboration on the subject [24]. The fertility defects we observed in our *Antxr2−/−* mice depend on the age of the mice at the time of analysis. Thus, the discrepancies between our results and the other study could be due to the age of the mice at the time of analysis, which was not specified in the *Liu et. al.* paper. In addition, the *Liu et. al.* group targeted the transmembrane domain of *Antxr2* for deletion. This targeting strategy may allow for the production of a secreted variant of Antxr2, which could have functional significance. For instance, our study demonstrates that the extracellular domain alone can influence MMP activity (Figure 7). Our strategy of targeting exon 1 for deletion results in the complete loss of Antxr2 protein expression (Figure 1).

The gradual accumulation of ECM proteins in the *Antxr2−/−* uterus suggested defective ECM remodeling, a process that should normally occur during each round of the estrus cycle. This prompted us to evaluate MMP activity in the *Antxr2−/−* mice. We discovered that ANTXR2 can be found in a complex with MT1-MMP (Figure 8) and that co-expression of ANTXR2 and MT1-MMP in 293T cells promotes activation of the MT1-MMP/MMP2 proteolytic cascade (Figure 7). Enhanced MMP2 processing from cells co-expressing ANTXRs and MT1-MMP (Figure 7) could be attributed to increased levels of MT1-MMP in those cells. At times we did observe increased MT1-MMP protein expression in 293T cells that were also expressing ANTXR2 (Figure 7F and Figure 8B), but this was not always the case (Figure 7C). It remains to be determined whether the ANTXRs increase steady state levels of MT1-MMP in cells and this will be the subject of future studies.

Our data leads us to hypothesize that ANTXR interaction with ECM components may facilitate multimerization and activation of a pericellular ANTXR/MT1-MMP complex. The fibrosis present in both the pregnant and nonpregnant uterus and cervix of *Antxr2−/−* mice may be the result of reduced Mt1-mmp activity in these tissues. In addition to its role in processing pro MMP2, MT1-MMP itself can degrade a number of ECM proteins including gelatin, fibronectin, vitronectin, fibrillar collagens and aggrecan [25]. It can also cleave a variety of other substrates, including cell surface receptors, growth factors, and cytokines [26]. We propose that in the absence of Antxr2, Mt1-mmp and Mmp2 proteolytic activities are diminished in the uterus and cervix. In support of this hypothesis, western blots on uterine lysates from *Antxr2−/−* mice demonstrated increased levels of pro MMP2 and a corresponding decrease in the levels of active Mmp2 in the tissue. It should be noted that while MT1-MMP is regarded as the main activator of MMP2, there are other pathways that regulate MMP2 activity. This is illustrated by the fact that zymographic analysis detected active MMP2 in tissues from *Mt1-mmp−/−* mice, albeit at greatly reduced levels [27]. Thus, in *Antxr2−/−* mice, it is likely that defective/reduced Mt1-mmp and Mmp2 activity resulted is an accumulation of type I collagen, type VI collagen, fibronectin and possibly other ECM proteins with each round of the estrus cycle. *Mt1-mmp−/−* mice have not been evaluated for reproductive defects since approximately 30% of the animals die before weaning with the remaining mutant mice dying between two to three months of age, however, it was noted that the *Mt1-mmp−/−* mice display no signs of sexual maturation [28].

The reproductive defects in female *Antxr2−/−* mice highlight the importance of the ANTXR/MT1-MMP complex for proper myometrial cell function. The myometrium has been demonstrated to express MT1-MMP [29] and MMP2 has been localized to the myometrium in the cycling uterus and during postpartum involution [16]. These reports point to myometrial cells as important mediators of ECM turnover in the remodeling uterus. The fact that accumulation of ECM proteins in the *Antxr2−/−* uterus coincides with the loss of myometrial cells suggests that a functional ANTXR/MT1-MMP complex is necessary for myometrial cells to effectively remodel the surrounding matrix.

Loss of the myometrium in the pregnant and non-pregnant *Antxr2−/−* uterus indicates that the ANTXR/MT1-MMP complex may also be essential for myometrial cell proliferation and viability. It is well established that the myometrium undergoes gradual changes during pregnancy, including a proliferative burst [30]. In the non-pregnant, sexually mature animal, myometrial cell proliferation is an integral part of the estrus cycle with the proliferative index peaking during proestrus [31]. It has recently been reported that MT1-MMP is a necessary cofactor for proper signaling through the PDGF-B/PDGFRβ axis in vascular smooth muscle cells [32]. Uterine myometrial cells have been demonstrated to express PDGFR, and treatment with PDGF induces a proliferative response in the cells [33]. Therefore, the PDGF signaling pathway may be an important growth factor that stimulates myometrial cell proliferation and survival during pregnancy and in the cycling uterus. It remains to be determined whether myometrial cell proliferation is impaired in the *Antxr2−/−* mice, but myometrial cell viablility is clearly affected in the animals and future studies will determine if Antxr2 regulation of MT1-MMP activity intersects with the PDGFR signaling pathways in the myometrium.

Patients with JHF and ISH, the human diseases caused by mutations in the *ANTXR2* gene, develop symptoms after birth and clinical features of the diseases include skin fibromas, gingival hypertrophy, joint contractures, osteoporosis and in the case of ISH, a failure to thrive [8], [11]. The skin fibromas are thought to form as a result of excessive ECM accumulation. Remarkably, the phenotype of the *Mt1-mmp−/−* mouse bears a strong resemblance to the symptoms exhibited by patients with JHF and ISH. Mt1-mmp has been demonstrated to have little or no role in embryonic development, however loss of expression in the mouse results in progressive impairment of postnatal growth and development affecting both the skeleton and soft connective tissue [27], [28], [34]. Similar to humans with JHF and ISH, aging in the *Mt1-mmp−/−* mice is associated with generalized fibrosis, progressive craniofacial dysmorphism, joint contractures, severe reduction of bone growth (ostopenia), reduced mobility, and a failure to thrive [27], [28]. Thus, our discovery that ANTXR2 positively regulates MT1-MMP activity could explain the phenoytpes associated with JHF and ISH. *Antxr2−/−* mice did not phenocopy JHF and ISH, nor did they phenocopy Mt1-mmp−/− mice. We documented that the activation of MT1-MMP is also regulated by ANTXR1 (Figure S4), therefore, in some tissues Antxr1 could be compensating for loss of Antxr2 in our mutant mice. This highlights the importance of evaluating the phenotypes associated with *Antxr1−/−;Antxr2−/−* mice.

It is also interesting to note that a recent paper reported that MT1-MMP cleaves the anthrax toxin binding moiety, protective antigen (PA), leading to shedding of PA proteolytic fragments from cell surfaces [35]. Since PA is a ligand of ANTXRs, that finding not only supports our discovery that ANTXR2 and MT1-MMP interact, but suggests that this interaction might negatively regulate the process of anthrax intoxication. Further investigation will help us understand this interaction.

While the mechanistic processes underlying ANTXR2/MT1-MMP interactions require further study, our research establishes a role for ANTXR2 as a regulator of MT1-MMP activity. We have discovered that ANTXR1 functions in a similar manner, which may explain the ECM accumulation observed in various organs of the *Antxr1−/−* mouse [9]. This novel mechanism of action for ANTXRs sheds light on the phenotypes associated with JHF and ISH and will inform future studies whether they are aimed at targeting anthrax intoxication or tumor growth and metastasis.

## Materials and Methods

### Generation of *Antxr2* knockout mice

Bacterial Artificial Chromosome RP23-162D22 (CHORI), containing the entire mouse *Antxr2* gene, was used as a template during BAC recombineering to construct a conditional *Anxtr2* targeting vector in which a single *loxP* site was inserted within the promoter region of the *ANTXR2* gene, a floxed neomycin cassette (NEO) was inserted within intron 1 for positive selection and a diptheria toxin A (DTA) cassette was inserted in place of exon 3 for negative selection. The BAC targeting construct was linearized with PI-SCE I, purified by phenol/choloroform extraction and electroporated into 129/SvJ embryonic stem (ES) cells by Columbia University's Herbert Irving Cancer Center Transgenic Mouse Facility. After G418 selection, four hundred ES cell clones were screened by Southern analysis to determine which clones had undergone homologous recombination. Briefly, gDNA isolated from ES cells was digested with BamHI and Southern blots were hybridized with a ^32^P-labeled probe to exon 3. This probe was designed to hybridize to a section of the gene outside the targeting vector homology arms in order to distinguish properly targeted recombination events from random integration. Four of the 400 ES cell clones screened had undergone proper targeting yielding a 4.4 kb band for the recombined allele and a 8 kb band for the wild-type allele (Figure S1B). PCR was also used to detect the presence of the single *loxP* site upstream of exon 1 (Figure S1B). Of these four ES cell clones, two were microinjected into host KV1 (129/Svj-C57B6 hybrid) blastocysts to generate chimeric animals. Mating the male chimeras with female C57BL/6 mice resulted in germline transmission of the *Antxr2 triloxP* allele to the F1 generation. Mice heterozygous for the *Antxr2 triloxP* allele were intercrossed to produce homozygous *Antxr2 triloxP* mice. *Antxr2+/−* mice were derived in two mating steps. First we mated male mice heterozygous for the *Antxr2 triloxP* allele with female *Ella-Cre* transgenic mice. The maternally derived *Cre* is more efficient at producing total germline excision of the *loxP1* and *loxP3* flanked DNA (i.e. deletion of exon 1 and NEO cassette) due to the presence of *Cre* in the oocyte. As this mating has the potential to produce mosaic offspring, genotyping was performed to detect the various recombination products and the *Cre* allele in order to identify mice that were heterozygous for both the *Antxr2* allele and the *Cre* allele (data not shown). To segregate the *Cre* allele, we next mated *Antxr2+/−*;*Cre* mice with wild type C57BL/6. Once we had obtained *Antxr2+/−* mice, we set-up intercrosses to produce *Antxr2−/−* mice.

### Genotyping

Mice were genotyped by PCR amplification of genomic DNA from tails. Primers for genotyping the conditional *Antxr2* allele (*Antxr2* floxed) were Forward 5′-CAGAACTCTAGGTCAGGGGC-3′ and Reverse 5′-CTTATGCCTCATCCCTCCGC-3′. This primer set yielded a 672 bp band to indicate the presence of the *loxP* site and a 600 bp band corresponding to the wild-type allele. Triplex PCR with three primers was used to detect knockout and wild-type *Antxr2* alleles simultaneously; a common Forward primer 5′-CGGTCACCCTGGAGCTATGC-3′ and allele-specific Reverse primers wild-type 5′-CTTATGCCTCATCCCTCCGC-3′ and knockout 5′- GAGGAAACGAGCTGCAGGTG-3′ were used. This primer set yielded a 316 bp band to indicate the presence of the *Antxr2* knockout allele and a 488 bp band corresponding to the wild-type allele.

### Ethics Statement and Animal Use

This study was conducted according to the National Institutes of Health *Guide for the Care and Use of Laboratory Animals*. Animal protocol, AAAD0577, was approved by the Columbia University Institutional Animal Care and Use Committee. Mice were housed under a 12 hr light cycle at 22°C. All *Antxr2−/−* mice and littermates were on a mixed C57BL/6-129SvJ background. Timed matings were performed by housing one male and two females in a cage. Each morning, females were evaluated for the presence of a plug and noon on the day a mating plug was detected was considered gestational day 0.5.

### Isolation of Mouse Embryonic Fibroblasts

Embryos were collected from the uteri of pregnant mice on gestational day 13.5. The heads and livers were removed and the carcasses were minced and trypsinized. Fibroblasts from the embryos were cultured in DMEM supplemented with 10% FBS and 50 mg/ml penicillin and streptomycin (GIBCO) in 5% CO_2_ at 37°C. gDNA isolated from embryo yolk sacs was used for genotyping PCR.

### Reverse Transcription PCR

Total RNA was isolated from MEFs using the RNeasy kit (Qiagen, Valencia, CA). First strand cDNA synthesis was performed using random hexamers and Superscript II reverse transcriptase (Invitrogen, Carlsbad, CA). PCR for mouse *ß-actin* and mouse *Antxr2* was performed using PCR primers as follows: mouse *Antxr2* exon1 Forward 5′-CTCTTGCAAAAAAGCCTTCG-3′ and Reverse 5′-TTCTTTGCCTCGTTCTCTGC-3′; mouse *Antxr2* exon2 Forward 5′-GTCTGGCAGTGTAGC-3′ and Reverse 5′-TTCTTTGCCTCGTTCTCTGC-3′; mouse *ß-actin* Forward 5′-CGAGGCCCAGAGCAAGAGAG-3′ and Reverse 5′-CTCGTAGATGGGCACAGTGTG-3′.

### Histologic Evaluation of Mouse Tissue

Analysis of the parturition defect was conducted using three *Antxr2+/+* and seven *Antxr2−/−* female mice. Reproductive tracts were isolated on GD18.5, fixed in 4% paraformaldehyde (PFA) and routinely processed for embedding in either OCT or paraffin. 5-µm serial sections were stained with H&E and Masson's Trichrome. See below for immunostaining. Reproductive tracts were isolated from nulliparous *Antxr2+/+* and *Antxr2−/−* mice at age 1 month to 15 months. At the time of collection, a small portion of each uterine horn was snap frozen in liquid nitrogen for immunoblotting analysis (see below). We analyzed tissue from three animals per genotype for each age group. The tissues were treated as specified above.

### Colorimetric IHC

For immunohistochemical studies evaluating Antxr2 expression in pregnant uterine tissue, fixed frozen 5-µm serial sections were post-fixed in acetone, blocked in phosphate-buffered saline (PBS) containing 3% bovine serum albumin and 2% rabbit serum (Sigma). Primary antibody goat anti-mouse Antxr2 (R&D) was incubated overnight at 4°C. Negative controls were left with blocking solution. Incubation with biotinylated secondary antibody rabbit anti-goat (Vector Laboratories) was performed for one hour at room temperature and followed by incubation with avidin and horseradish-peroxidase conjugated biotin in PBS (Vectastain Standard ABC Elite kit, Vector Laboratories). The color reaction was performed using DAB (diaminobenzidine tetrahydrochloride), the peroxidase substrate (Vector Laboratories). Tissues were counterstained with hematoxylin (Fisher).

### Immunofluorescent IHC

Immunostaining was performed as described above until application of primary and secondary antibodies. Primary antibodies used were: mouse anti-∝SMACy3 (Sigma), biotinylated rabbit anti-type VI collagen (Rockland), rabbit anti-type I collagen (Millipore), rabbit anti-fibronectin (Abcam), rat anti-mouse CD31 (BD Pharminogen), rat anti-endomucin (Santa Cruz), goat anti-lyve-1 (R&D), rat anti-mouse F4/80 (Abcam). Sections were incubated with Alexa Fluor tagged secondary antibodies (Molecular Probes), which were specific to each primary antibody. DAPI (4, 6-diamidino-2-phenylindole) (Sigma) was used to visualize nuclei. Negative controls were treated with secondary antibody alone. Images were obtained on Nikon ECLIPSE E 800 microscope (Nikon Inc.).

### Serum Progesterone Measurements

Progesterone levels were measured in the sera of mice on gestational days 15.5 and 18.5. Sera were collected from three *Antxr2+/+* mice and five *Antxr2−/−* mice. Blood was drawn via cardiac puncture, allowed to clot at room temperature for 30 minutes and centrifuged to remove red blood cells. The sera were stored at −80°C until time of analysis. Serum progesterone levels were measured using a mouse progesterone ELISA kit (Cusabio Biotech Co.) following manufacturer instructions.

### ANTXR2 Gene Silencing and Cell Surface Receptor Expression Analysis

ANTXR2 gene silencing in HUVEC cell lines has been described [6]. Flow cytometry analysis of ANTXR2 expression on the cell surface has been described [36].

### DNA Constructs

ANTXR2-GFP and ANTXR2-vWF constructs have been described [37]. ANTXR1-GFP and ANTXR1-vWF constructs have been described [37], [38]. All of these constructs were engineered into retroviral vector pHyTCX for the experiments described herein. Wild-type MT1-MMP and C-terminally truncated MT1-MMP (MT1-ΔC) constructs have been described [39].

### Transfections and Gelatin Zymography

Gelatin Zymography analysis was performed as previously described [40], [41]. 5×10^4^ 293Ts were seeded in 400 ul of DMEM with 10% fetal bovine serum in a 24 well plate. Cells were transfected with Effectene (Qiagen) according to the manufacturer's protocol. After transfection, cells were washed with PBS, and cultured in DMEM with 5% fetal bovine medium (the source of pro MMP2). After 16–24 hours, the condition medium was harvested and cleared by centrifugation at 12,000 rpm for 10 minutes and subjected to analysis by SDS-substrate gel electrophoresis (zymography) under non-denaturing conditions in 8.0% SDS-polyacrylamide gels impregnated with 1 mg/ml gelatin as previously described [40], [41]. The gels were incubated at 37°C overnight in 50 mM Tris (pH 7.5), 5 mM CaCl_2_, 1 mM ZnCl_2_ and stained with Coomassie Brilliant Blue R25. Destained gel images were captured by Kodak EL Logic 100 Imaging System. For MEF and 293T zymography, experimental samples were tested in duplicate. For HUVEC zymography, all of the experimental samples were tested in quadruplicate. All of the experiments were repeated twice. ImageJ 1.45 s (NIH) was used to quantify zymography band intensities.

### Tissue lysate preparation and immunoblotting

Uterine tissues were homogenized on ice in 500 mL RIPA buffer (50 mM Tris-HCl, pH 7.5, 10 mM EDTA, 150 mM NaCl, 1% Nonidet P-40, and protease inhibitor cocktail). Homogenized lysate was clarified by centrifugation at 12,000 rpm at 4°C for 10 minutes. Protein concentration was determined using Bradford reagent (BioRad). Lysates containing 10 µg of protein were electrophoresed in the appropriate percentage SDS-polyacrylamide gel (6% for type I collagen, type VI collagen, fibronectin; 10% for MMP2, MT1-MMP, MT1-ΔC, ANTXR2-GFP, and ANTXR2-vWF). Protein was transferred to nitrocellulose by electroblotting and then blocked for 1 hour at 22°C in PBST (1×PBS, 0.2% Tween) containing 3% bovine serum albumin. Blots were incubated with appropriate primary antibodies in blocking solution overnight at 4°C. Antibodies used were biotinylated rabbit anti-type VI collagen (Rockland), rabbit anti-type I collagen (Millipore), rabbit anti-fibronectin (Abcam), rabbit anti-MMP2 (Abcam), rabbit anti-MT1-MMP (Epitomics), goat anti-ANTXR2 (R&D). The blots were washed three times for 10 minutes each in PBST and incubated in the appropriate HRP secondary antibodies for 1 hour at 22°C. The blots were washed as above and then incubated for 5 minutes in enhanced chemiluminescence reagents (Fisher) and exposed to film (Kodak).

### Immunocytochemistry

To visualize Mt1-mmp and Antxr2 on cell surfaces, MEFs were seeded on gelatin-coated coverslips in 24 well plates. The next day cells were washed twice with ice cold PBS and stained with rabbit anti-MT1-MMP (Epitomics) and goat anti-Antxr2 (R&D) for one hour at 4°C. The cells were washed three times in ice cold PBS and fixed in 4% PFA for 10 minutes at room temperature. After fixation, the cells were incubated in PBS containing 3% bovine serum albumin and 2% donkey serum for 30 minutes at room temperature and then stained with donkey anti-rabbit alexa fluor 488 and donkey anti-goat alex fluor 594 for 30 minutes at room temperature. Following three washes with PBS, coverslips were mounted in Vectashield containing DAPI. Images were obtained on Nikon ECLIPSE E 800 microscope. To reveal colocalization of the two proteins, the images were processed and merged in Adobe PhotoShop software.

### Immunoprecipitation

Transfected cells were lysed in RIPA buffer (50 mM Tris-HCl, pH 7.5, 10 mM EDTA, 150 mM NaCl, 1% Nonidet P-40, and protease inhibitor cocktail) for 30 minutes at 4°C. Cell extracts were cleared by centrifugation at 12,000 rpm for 10 minutes and the supernatant was incubated at 4°C with goat anti-ANTXR2 (R&D) for 2 hours. Immune complexes were immobilized on protein-A/G beads for 3 hours, washed three times with lysis buffer, and subjected to Western-blotting analysis with rabbit anti MT1-MMP antibody (Epitomics).

### Statistical analysis

Statistical significance was evaluated using the unpaired Student's *t* test with *P* value less than .05 considered statistically significant.

## Supporting Information

Figure S1 (A) Diagram of the first three exons of the *Antxr2* wild-type allele, the targeting vector, the *triloxP* allele in which a *loxP* site (arrowhead) was inserted upstream of exon 1 and a floxed *Neo* cassette was inserted within intron 1, and the knockout allele. The red box under exon 3 indicates the external probe used for Southern Blot analysis. The green arrows represent PCR primers used to detect the single *loxP* site upstream of exon 1. (B) Upper panel - Southern blot analysis of properly targeted ES cells. The wild-type allele is 8.174 Kb and the *TriloxP* allele is 4.4 kb. Lower panel - PCR analysis on gDNA to detect the *loxP* site upstream of exon 1. The 672 bp band represents the *loxP* allele and the 600 bp band represents the wild-type allele. (C) Masson's trichrome staining of *Antxr2+/+* and *Antxr2−/−* ovaries isolated on GD18.5 did not reveal differences in collagen content. CL, corpeus luteum. Scale bars, 400 µm. (D) ELISA analysis of sera from *Antxr2+/+* and *Antxr2−/−* mice on GD15.5 and 18.5 revealed that serum progesterone levels declined as the animals approached term (GD19). Sera from three *Antxr2+/+* mice and five *Antxr2−/−* mice were analyzed. The graph presents the mean ± the standard deviation. *P*>0.2 when comparing *Antxr2+/+* and *Antxr2−/−* progesterone levels at either time point.
(TIF)

Figure S2 Masson's Trichrome staining did not reveal differences in collagen content between *Antxr2+/+* and *Antxr2−/−* ovaries isolated from three-month-old animals or six-month-old animals. 3 month scale bars, 150 µm. 6 month scale bars, 200 µm.
(TIF)

Figure S3 Immunofluorescent staining of uterine tissue isolated from ten-month-old mice demonstrated increased type I collagen (green color), type VI collagen (red color) and fibronectin (red color) deposition in the *Antxr2−/−* tissue. L, uterine lumen. DAPI (blue color) is used for nuclear staining. Scale bars, 150 µm.
(TIF)

Figure S4 (A) Zymographic analysis of conditioned medium from 293T cells transfected with empty vector (lane 1), MT1-MMP (lane 2), ANTXR1-GFP (lane 3), ANTXR1-vWF (lane 4), ANTXR2-GFP (lane 5), MT1-MMP and ANTXR1-GFP (lane 6), MT1-MMP and ANTXR1-vWF (lane 7), MT1-MMP and ANTXR2-GFP (lane 8), MT1-ΔC (lane 9), MT1-ΔC and ANTXR1-GFP (lane 10), or MT1-ΔC and ANTXR1-vWF (lane 11), or MT1-ΔC and ANTXR2-GFP (lane 12) revealed that co-expression of either MT1-MMP or MT1-ΔC and ANTXR1-GFP or ANTXR1-vWF led to enhanced pro MMP2 activation over expression of either MT1-MMP or MT1-ΔC alone. Table under the zymogram represents densitometric quantification of the pro and active MMP2 bands. Numbers are in percentile of relative intensity in relation to the empty vector control, lane 1. (B) Zymographic analysis of conditioned medium from 293T cells co-expressing MT1-ΔC and varying concentrations of ANTXR2-GFP or ANTXR2-vWF revealed that MT1-ΔC activity is dependent on ANTXR2 expression levels. Table under the zymogram represents densitometric quantification of the pro and active MMP2 bands. Numbers are in percentile of relative intensity in relation to the empty vector control, lane 1. For each zymogram panel, a representative of two independent experiments is shown.
(TIF)
