# Supplementary figures and images for: Anthrax Toxin Receptor 2 Functions in ECM Homeostasis of the Murine Reproductive Tract and Promotes MMP Activity
Source: PLoS One. 2012 Apr 17;7(4):e34862. doi: 10.1371/journal.pone.0034862 (PMC3328497; doi:10.1371/journal.pone.0034862)

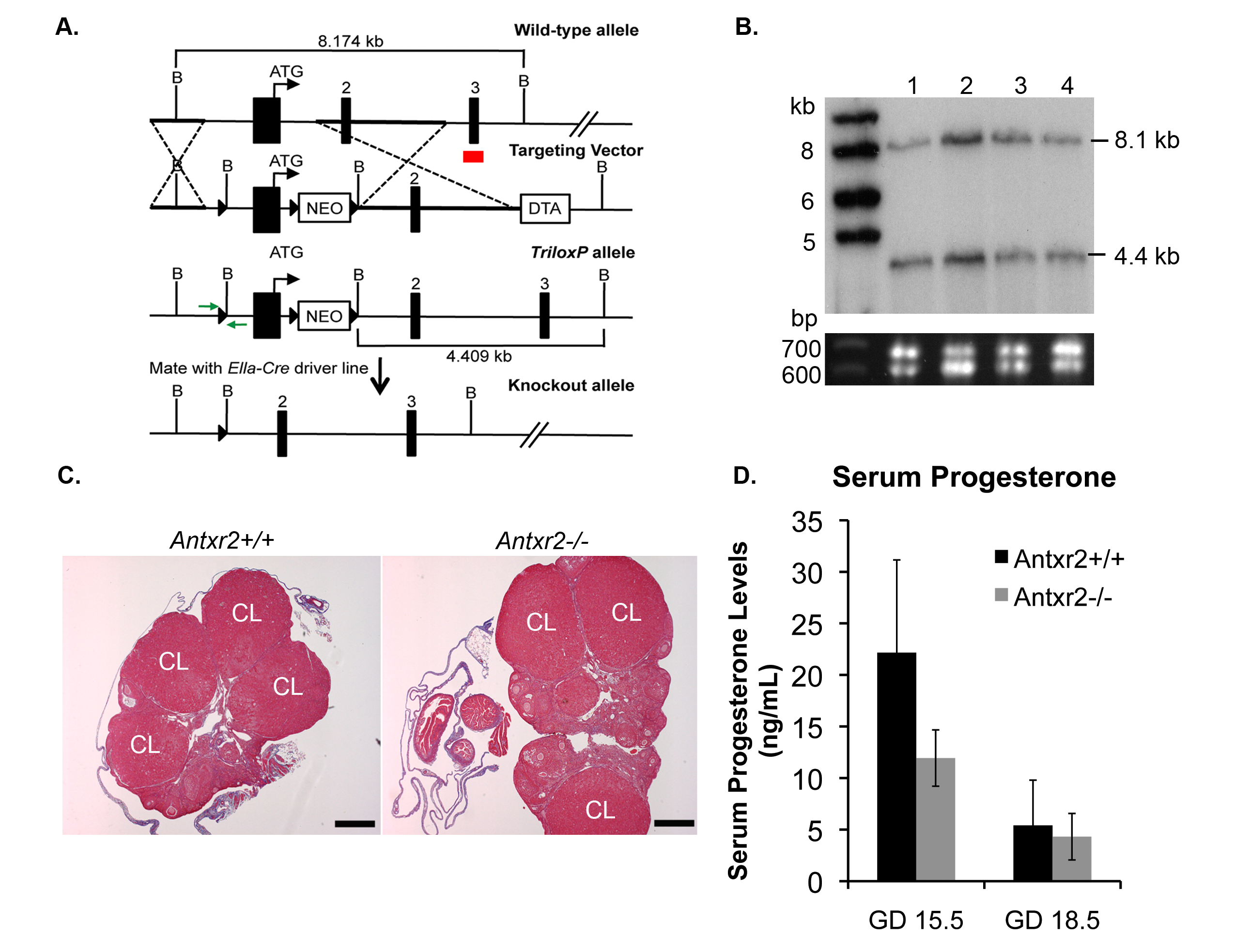

Supplement: Figure S1 — (A) Diagram of the first three exons of the Antxr2 wild-type allele, the targeting vector, the triloxP allele in which a loxP site (arrowhead) was inserted upstream of exon 1 and a floxed Neo cassette was inserted within intron 1, and the knockout allele. The red box under exon 3 indicates the external probe used for Southern Blot analysis. The green arrows represent PCR primers used to detect the single loxP site upstream of exon 1. (B) Upper panel - Southern blot analysis of properly targeted ES cells. The wild-type allele is 8.174 Kb and the TriloxP allele is 4.4 kb. Lower panel - PCR analysis on gDNA to detect the loxP site upstream of exon 1. The 672 bp band represents the loxP allele and the 600 bp band represents the wild-type allele. (C) Masson's trichrome staining of Antxr2+/+ and Antxr2−/− ovaries isolated on GD18.5 did not reveal differences in collagen content. CL, corpeus luteum. Scale bars, 400 µm. (D) ELISA analysis of sera from Antxr2+/+ and Antxr2−/− mice on GD15.5 and 18.5 revealed that serum progesterone levels declined as the animals approached term (GD19). Sera from three Antxr2+/+ mice and five Antxr2−/− mice were analyzed. The graph presents the mean ± the standard deviation. P>0.2 when comparing Antxr2+/+ and Antxr2−/− progesterone levels at either time point. (TIF) [file pone.0034862.s001.tif]

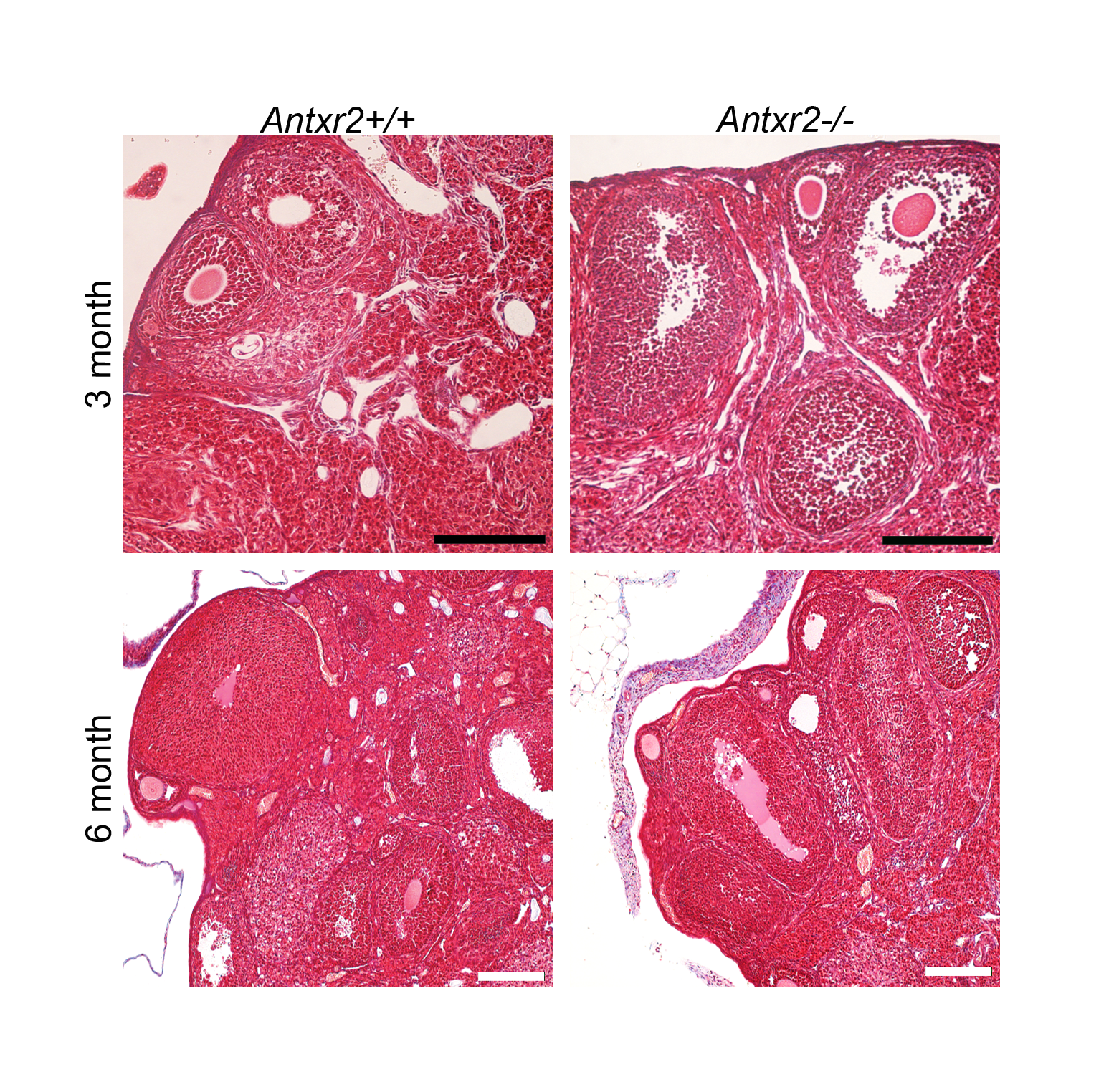

Supplement: Figure S2 — Masson's Trichrome staining did not reveal differences in collagen content between Antxr2+/+ and Antxr2−/− ovaries isolated from three-month-old animals or six-month-old animals. 3 month scale bars, 150 µm. 6 month scale bars, 200 µm. (TIF) [file pone.0034862.s002.tif]

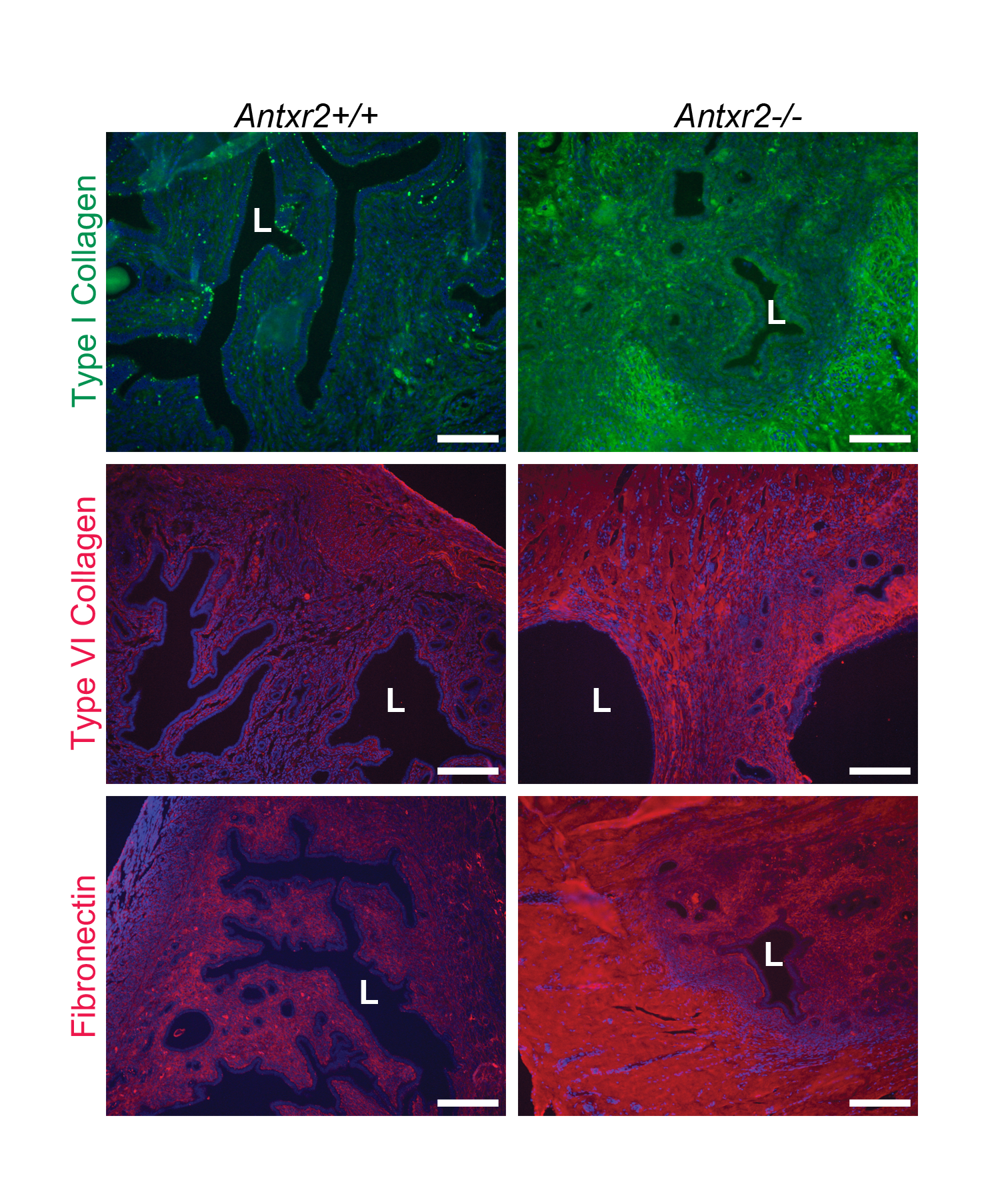

Supplement: Figure S3 — Immunofluorescent staining of uterine tissue isolated from ten-month-old mice demonstrated increased type I collagen (green color), type VI collagen (red color) and fibronectin (red color) deposition in the Antxr2−/− tissue. L, uterine lumen. DAPI (blue color) is used for nuclear staining. Scale bars, 150 µm. (TIF) [file pone.0034862.s003.tif]

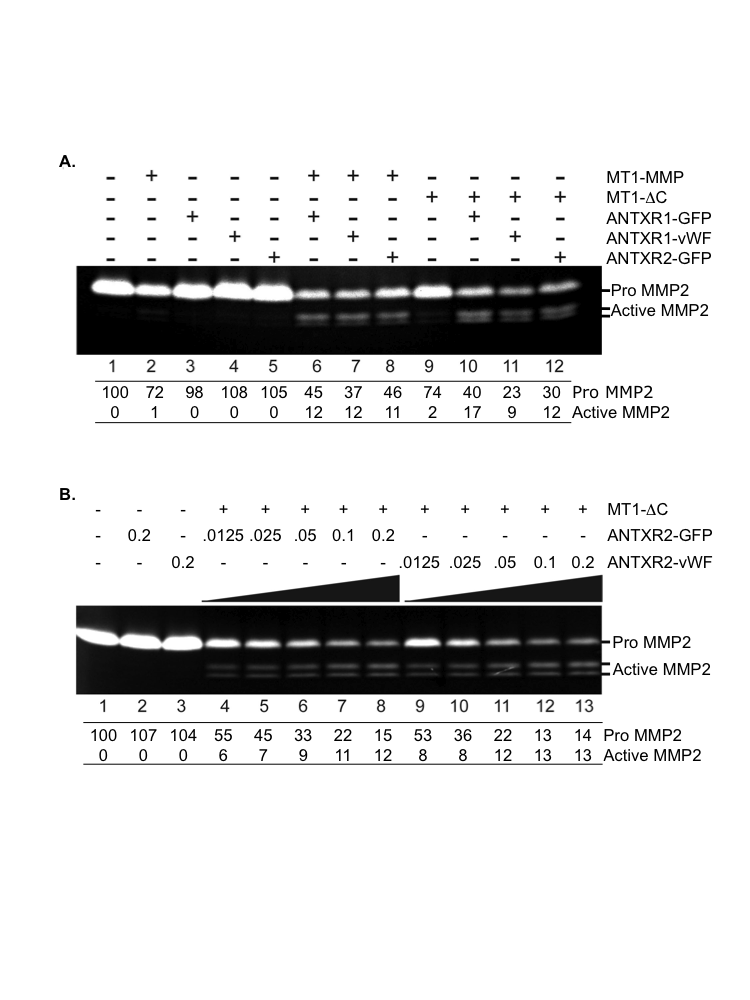

Supplement: Figure S4 — (A) Zymographic analysis of conditioned medium from 293T cells transfected with empty vector (lane 1), MT1-MMP (lane 2), ANTXR1-GFP (lane 3), ANTXR1-vWF (lane 4), ANTXR2-GFP (lane 5), MT1-MMP and ANTXR1-GFP (lane 6), MT1-MMP and ANTXR1-vWF (lane 7), MT1-MMP and ANTXR2-GFP (lane 8), MT1-ΔC (lane 9), MT1-ΔC and ANTXR1-GFP (lane 10), or MT1-ΔC and ANTXR1-vWF (lane 11), or MT1-ΔC and ANTXR2-GFP (lane 12) revealed that co-expression of either MT1-MMP or MT1-ΔC and ANTXR1-GFP or ANTXR1-vWF led to enhanced pro MMP2 activation over expression of either MT1-MMP or MT1-ΔC alone. Table under the zymogram represents densitometric quantification of the pro and active MMP2 bands. Numbers are in percentile of relative intensity in relation to the empty vector control, lane 1. (B) Zymographic analysis of conditioned medium from 293T cells co-expressing MT1-ΔC and varying concentrations of ANTXR2-GFP or ANTXR2-vWF revealed that MT1-ΔC activity is dependent on ANTXR2 expression levels. Table under the zymogram represents densitometric quantification of the pro and active MMP2 bands. Numbers are in percentile of relative intensity in relation to the empty vector control, lane 1. For each zymogram panel, a representative of two independent experiments is shown. (TIF) [file pone.0034862.s004.tif]
